# Multi-omics and single-cell approaches reveal molecular subtypes and key cell interactions in hepatocellular carcinoma

**DOI:** 10.3389/fphar.2025.1605162

**Published:** 2025-05-22

**Authors:** Xueqing Zou, Yongmei Wang, Mingyuan Luan, Yizheng Zhang

**Affiliations:** ^1^ Department of Anesthesiology, The Affiliated Hospital of Qingdao University, Qingdao, Shandong, China; ^2^ Department of Pathology and Neuropathology, University Hospital and Comprehensive Cancer Center Tübingen, Tübingen, Germany; ^3^ Breast Disease Center, The Affiliated Hospital of Qingdao University, Qingdao, Shandong, China

**Keywords:** hepatocellular carcinoma, multi-omics, tumor microenvironment, single-cell transcriptomics, tumor heterogeneity, precision oncology

## Abstract

**Introduction:**

Hepatocellular carcinoma is a highly aggressive and heterogeneous malignancy with limited understanding of its heterogeneity.

**Methods:**

In this study, we applied ten multi-omics classification algorithms to identify three distinct molecular subtypes of HCC (C1–C3). To further explore the immune microenvironment of these molecular subtypes, we leveraged single-cell transcriptomic data and employed CIBERSORTx to deconvolute their immune landscape.

**Results:**

Among them, C3 exhibited the worst prognosis, whereas C1 and C2 were associated with relatively better clinical outcomes. Patients in the C3 group exhibited a high burden of copy number variations, mutation load, and methylation silencing. Our results revealed that compared to C1 and C2, C3 had a lower proportion of hepatocytes but a higher proportion of cholangiocytes and macrophages. Through analyses of hepatocyte, cholangiocyte, and macrophage subpopulations, we characterized their functional states, spatial distribution preferences, evolutionary relationships, and transcriptional regulatory networks, ultimately identifying cell subpopulations significantly associated with patient survival. Furthermore, we identified key ligand-receptor interactions, such as APOA1-TREM2 and APOA2-TREM2 in hepatocyte-macrophage crosstalk, and VTN-PLAUR in cholangiocyte-macrophage communication.

**Discussion:**

Finally, we employed machine learning methods to construct a prognostic model for HCC patients and identified novel potential compounds for high risk patients. In summary, our novel multi-omics classification of HCC provides valuable insights into tumor heterogeneity and prognosis, offering potential clinical applications for precision oncology.

## 1 Introduction

Hepatocellular carcinoma (HCC) is one of the most aggressive and fatal malignancies worldwide, with a rising incidence, particularly in the Asia-Pacific region where it is commonly associated with chronic hepatitis B (Forner et al., 2018; Bray et al., 2018; Yang et al., 2019). In recent years, numerous novel therapeutic strategies targeting hepatocellular carcinoma have been proposed and actively investigated (Wang et al., 2024; Guo et al., 2023). Despite advances in early diagnosis and treatment strategies, the prognosis remains poor, largely due to the high recurrence rates and tumor heterogeneity inherent in HCC (Yang et al., 2019). The complex biological and molecular landscape of HCC leads to variable clinical outcomes, with patients at the same clinical stage exhibiting vastly different responses to therapy (Yang et al., 2019; Villanueva, 2019). This heterogeneity complicates effective treatment strategies and underscores the urgent need for improved classification systems that can guide precision medicine.

Traditional approaches to HCC classification have largely relied on histopathological features. These methods often fail to capture the full complexity of HCC’s molecular landscape. Molecular subtyping has emerged as a competent method with huge potential in addressing tumor heterogeneity and determining the applicability of precision treatment for patients with HCC. Over the past decade, considerable research has been conducted to develop molecular subtype systems based on RNA sequencing data. For example, classifications have been proposed by Boyault et al. (G1–G6) (Boyault et al., 2007), Chiang et al. (five subclasses) (Chiang et al., 2008), Hoshida et al. (S1–S3) (Hoshida et al., 2009), Désert et al. (four subclasses) (Désert et al., 2017), and Yang et al. (C1–C3) (Yang et al., 2020). While these molecular subtype systems have been proposed for HCC, most have been developed using bulk tissue samples and do not fully account for the spatial and cellular heterogeneity present within the tumor. The combination of single-cell analysis with multi-omics profiling presents a promising strategy to refine these subtyping systems, enabling the identification of more precise molecular subtypes that reflect the true complexity of HCC.

In this study, we constructed a comprehensive multi-omics molecular classification system for HCC by integrating multi-omics and scRNA-seq data. Through the analysis of molecular and immune profiles of patients, we partially revealed the differences in the tumor microenvironment (TME) among different subtypes, which may help explain the prognostic disparities among patients (Wu et al., 2023). This integrated approach will provide a deeper understanding of the underlying biology of HCC, with the potential to inform personalized treatment strategies and improve patient outcomes.

## 2 Methods

### 2.1 Data collection

Gene expression data (raw counts), methylation data, copy number variation (CNV) data, mutation data, clinical data, and sample information of TCGA-LIHC cohort were downloaded from The Cancer Genome Atlas (TCGA) website (https://portal.gdc.cancer.gov/repository). Raw counts were transformed into TPM values for subsequent analysis.

The raw data for single-cell sequencing of HCC (GSE156625) (Sharma et al., 2020) were downloaded from SRA Run Selector (https://www.ncbi.nlm.nih.gov/Traces/study/?acc=PRJNA658535&o=acc_s%3Aa).

### 2.2 Multi-platform integrative clustering using MOVICS

To identify the subtypes of HCC by integrating multi-omics data from various molecular platforms, we utilized the MOVICS package (version 0.99.17) (Lu et al., 2021) in R, which incorporates ten machine learning based algorithms: Consensus Clustering, iClusterBayes, iClusterPlus, Similarity Network Fusion, Non negative Matrix Factorization, k-means clustering, Network based Stratification, Multi-Omics Factor Analysis, PINSPlus, and CIMLR. The mRNA expression (Transcripts per million, TPM) data, DNA methylation data, CNV data, mutation data from TCGA-LIHC were used as input.

For the mRNA data, probes with >75% missing values were excluded. The mRNA data were log2 transformed. Genes with a standard deviation <1.0 across all tumor samples were filtered out from the mRNA datasets.

Methylation data were processed using the ChAMP package (version 2.21.1) (Tian et al., 2017) in R. Methylation probes from the Illumina Infinium 450k arrays with >20% missing values were removed, along with probes corresponding to SNPs and sex chromosomes. For the remaining methylation probes, NA values were imputed using the champ.impute function, and correction was performed using the champ.BMIQ function. Finally, the median absolute deviation (MAD) of the methylation values was calculated, and the top 1,000 methylation probes with the highest MAD values were selected as input for MOVICS.

CNV data were analyzed using GISTIC 2.0 (Mermel et al., 2011). We used the CN values of significantly amplified and deleted regions from new_all_lesions.conf_99 as input for MOVICS.

For the mutation data, we used a mutation matrix of driver mutations as input. The information on driver mutations in TCGA-LIHC was obtained from the Broad Institute’s GDAC Firehose platform (https://gdac.broadinstitute.org/runs/analyses__latest/data/LIHC/20160128/) and was identified using the MutSig algorithm (version 2.0) (Network, 2011).

It is widely acknowledged that the optimal number of clusters should be sufficiently small to minimize noise, yet large enough to preserve essential biological information. Therefore, we utilized the “getClustNum” function from the MOVICS package to estimate the number of subtypes. This estimation was based on multiple criteria, including the Clustering Prediction Index (CPI), Gap Statistics, and Silhouette Score. We also considered the molecular characteristics of the subgroups across different clustering numbers. Based on these integrative evaluations, we ultimately determined that the samples could be categorized into three distinct subtypes.

### 2.3 Molecular characteristics of multi-omics subtypes

For the transcriptomic data, we applied the ssGSEA algorithm to calculate the enrichment score of each pathway in each sample, thereby assessing the activity level of each pathway in individual samples.

To identify the molecular features of each multi-omics subtype, we modified the source code of the clinicalEnrichment function in the maftools package (Mayakonda et al., 2018), enabling its application to copy number data and methylation data.

For the identification of subtype specific driver mutations, we utilized the clinicalEnrichment function from the maftools package and visualized the results using plotEnrichmentResults in the maftools package.

For subtype specific CNV data, we used the discrete values from the new_all_lesions.conf_99 file in the GISTIC results as input. First, we converted these values into a matrix representing amplification/deletion or no amplification/deletion, followed by analysis using the modified clinicalEnrichment source code, and visualized the results with plotEnrichmentResults in the maftools package. The burden of copy number loss or gain was calculated as the total number of genes with copy number changes at the focal and arm levels.

For the methylation data, we identified subtype specific methylation silenced genes. We applied a previously published method to identify methylation silenced genes (Network et al., 2017). The complete workflow is shown in Supplementary Figure S1. Initially, we excluded SNPs and DNA methylation probes associated with the X or Y chromosomes. Then, we focused on probes situated within the promoter region, defined as 1,500 bp upstream and downstream of the transcription start site. Next, we removed CpG sites that were methylated in normal tissues, with an average β-value exceeding 0.2. Following this, we applied a β-value threshold of >0.3 to classify the DNA methylation data, designating sites as methylated, while excluding CpG sites methylated in fewer than 5% of tumor samples. For each probe/gene pair, we implemented the following procedure: 1) tumor samples were categorized into methylated (β ≥ 0.3) and unmethylated (β < 0.3) groups; 2) the mean expression levels for both groups were computed; 3) the standard deviation of the expression values in the unmethylated group was determined. Subsequently, we selected probes where the mean expression in the methylated group was at least 1.64 standard deviations lower than the mean expression in the unmethylated group. A tumor sample was marked as epigenetically silenced if: a) it belonged to the methylated group and b) the expression of the corresponding gene was lower than the average expression in the unmethylated group. When multiple probes were linked to the same gene, samples that were labeled as epigenetically silenced in more than half of the probes were also classified as epigenetically silenced at the gene level. Finally, we quantified the methylation silencing burden for each patient by calculating the number of methylation silenced genes in each sample.

### 2.4 Single-cell RNA-Seq data processing

We utilized the human HCC single-cell dataset from GSE156337 (Sharma et al., 2020). A detailed overview of the entire single-cell analysis workflow is provided in Supplementary Figure S2. We processed the single-cell data using Scanpy (version 1.9.6) (Wolf et al., 2018) in python and computed the proportions of counts for mitochondrial genes for all cells using scanpy.pp.calculate_qc_metrics. We applied the following filtering criteria (Forner et al., 2018): cells with fewer than 200 expressed genes (Bray et al., 2018); genes detected in fewer than 30 cells (Yang et al., 2019); cells with more than 5% of their counts from mitochondrial genes (Wang et al., 2024); cells with total counts per cell greater than 4000 (Guo et al., 2023); doublet cells were filtered using Scrublet (version 0.2.3) with default parameters, except that a fixed random seed (random_state = 112) was set (Scrublet et al., 2019). The total expression levels of each cell were normalized and log transformed using the scanpy.pp.normalize_total and scanpy.pp.log1p functions. The top 3,000 highly variable genes were selected using scanpy.pp.highly_variable_genes, and the expression values were scaled to a maximum of 10 using scanpy.pp.scale. The scVI (single-cell Variational Inference) package (version 0.20.3) (Lopez et al., 2018) was applied to correct for batch effects. Subsequently, dimensionality reduction was performed on the cells' representations in the latent space using scanpy.pp.neighbors and scanpy.tl.umap, followed by visualization using UMAP (Uniform Manifold Approximation and Projection). Cell subpopulations were identified using scanpy.tl.leiden. First, we use PTPRC, CD3D, CD8A, CD8B, CD4, FOXP3, NKG7, GNLY, MS4A1, and CD79A to determine whether a cell subpopulation belongs to the lymphoid lineage. CD14, FCGR3A, XCR1, LAMP3, C1QA, CD68, CD163, and TPSAB1 are used to identify cells of the myeloid lineage. PECAM1 is used to identify endothelial cells. COL1A1 and FAP are used to identify fibroblasts. ACTA2 is used to identify hepatic stellate cells (HSCs). We then performed scanpy.tl.leiden separately on each of these groups and classified them into different cell subpopulations based on distinct markers and functional scores. Subsequently, we used the reticulate package (version 1.34.0) (Kevin et al., 2024) in R to call the anndata package (version 0.10.5.post1) (Virshup et al., 2024) in Python to read the h5ad file generated by Scanpy and converted it into Seurat (version 5.0.1) (Hao et al., 2024) format. We obtained the HALLMARK database using the.get_gmt function from gseapy (version 1.1.3) (Fang et al., 2023) and performed HALLMARK gene set scoring using the tl.score_genes function from the scanpy package. Based on our identified cell subtypes, we applied the FindAllMarkers function from the Seurat package to identify the expression characteristics of each cell subgroup. Subsequently, we calculated the average expression of these markers across all cell types to generate the marker gene matrix, which was then used as the input for CIBERSORTx (https://cibersortx.stanford.edu/) (Newman et al., 2019). Monocle3 R package (https://cole-trapnell-lab.github.io/monocle3/) was used for pseudotime trajectory analysis (Qiu et al., 2017). All pseudotime associated gene modules were subjected to KEGG enrichment analysis using DAVID (Bt et al., 2022) (https://davidbioinformatics.nih.gov/home.jsp) to elucidate their functions. For functional analysis of each cell subpopulation, we first identified cell markers using FindAllMarkers, followed by KEGG pathway enrichment analysis through GSEA using the ClusterProfiler (Xu et al., 2024) R package. For survival analysis based on cell proportions from the CIBERSORTx results, we used the survival package (Therneau, 2024) in R. Samples were stratified into groups based on the median value, and significance was assessed using the log-rank test.

### 2.5 Single-cell RNA velocity analysis

To analyze RNA velocity, we utilized scVelo (version 0.3.3) (Bergen et al., 2020) following a standardized workflow. First, we used the output from CellRanger as input and processed the aligned single-cell RNA sequencing data with velocyto.py (La Manno et al., 2018) to generate a loom file for each sample. Each loom file contained spliced and unspliced transcript counts, which were subsequently integrated with the corresponding single-cell sequencing data. Next, we processed these data using the default pipeline of scVelo and employed the stochastic model to infer transcriptional dynamics.

### 2.6 Gene regulatory network analysis

We performed Single-cell regulatory network inference and clustering (SCENIC) analysis using pySCENIC (v0.12.1) to reconstruct transcriptional regulatory networks. The relationships between transcription factors (TFs) and their downstream target genes were identified based on hg38_10kbp_up_10kbp_down_full_tx_v10_clust.genes_vs._motifs.rankings.feather and motifs-v10nr_clust-nr.hgnc-m0.001-o0.0.tbl, following the guidelines provided in the pySCENIC documentation (https://github.com/aertslab/pySCENIC). The analysis was conducted using the raw count matrix as input, with default parameters. Initially, co-expression modules were constructed, and the regulatory relationships between TFs and their target genes were inferred using GRNBoost. Next, RcisTarget was employed to identify regulons—TFs with direct target genes—by integrating motif enrichment analysis. Finally, the regulon activity in individual cells was quantified using AUCell. For the analysis of transcription factor regulatory patterns, we used the NMF package (version 0.26) (Gaujoux and Seoighe, 2010) in R. Rank estimation for NMF was performed using the nmfEstimateRank function with the Lee method (method = “lee”) over a rank range of one–6, with 30 runs (nrun = 30) and a fixed seed (seed = 10). The cophenetic correlation and silhouette consensus scores were extracted to assess clustering stability. We performed NMF using the nmf function from the NMF package with the optimal rank determined based on cophenetic correlation and silhouette consensus scores. We then extracted the basis matrix to analyze TFs expression patterns.

### 2.7 Cell-cell communication analysis

We used the CellChat (Jin et al., 2025) algorithm (https://github.com/jinworks/CellChat, version 2.1.2) to elucidate cell-cell interactions. Following the standard workflow, we transferred the count data from Seurat into CellChat for further analysis. Utilizing CellChat’s built-in ligand-receptor database, we inferred potential intercellular communications. The communication probabilities and associated pathways were then computed using the “computeCommunProb” and “computeCommunProbPathway” functions.

### 2.8 Construction of the prognostic scoring system

To more accurately assess the prognosis of patients with different subtypes and enhance its applicability in clinical practice, we developed a prognostic scoring system. First, we performed univariate Cox regression analysis to identify genes significantly associated with prognosis (P < 0.01). Subsequently, we split the TPM expression data of the TCGA-LIHC cohort into a 70% training set and a 30% test set. Then we applied least absolute shrinkage and selection operator (LASSO) Cox regression analysis with 10 fold cross validation using the “glmnet” package in R to select the optimal prognostic gene signatures associated with HCC (Friedman et al., 2010). The prognostic score was calculated based on the relative expression levels of the selected gene signatures and their corresponding Cox regression coefficients, using the following formula:

$$\text{risk score}=\sum_{i=1}^{n}\left({\text{Coef}}_{i}\times {\text{Expr}}_{i}\right)$$

where Coefi represents the LASSO Cox coefficient of gene signature i, and Expri denotes the expression level of the gene in patient i. Based on the median prognostic score, patients were classified into high risk and low risk groups. To further evaluate the predictive capability of the prognostic score, we performed univariate and multivariate Cox regression analyses incorporating several key clinical features from the TCGA dataset.

### 2.9 Potential therapeutic sensitivity assessment

To further evaluate the differences in therapeutic sensitivity among different subtypes of HCC patients, we first collected several therapy specific signatures, including immune inhibited oncogenic pathways, signatures related to epidermal growth factor receptor (EGFR) targeted therapy, and signatures associated with radiotherapy, from a previous study (Hu et al., 2021) (Supplementary Table S7).

We assessed their levels using the ssGSEA method in GSVA R package (Sonja et al., 2013). Subsequently, we screened potential sensitive drugs for patients with different risk levels. The expression profile data of human cancer cell lines (CCLs) were downloaded from the Broad Institute’s Cancer Cell Line Encyclopedia (CCLE) (Ghandi et al., 2019). Drug sensitivity data for CCLs were obtained from the Cancer Therapeutics Response Portal (CTRP) version 2.0 (https://portals.broadinstitute.org/ctrp) and the PRISM Repurposing dataset (19Q4; https://depmap.org/portal/prism/). This dataset provides area under the curve (AUC) values as a measure of drug sensitivity, where lower AUC values indicate increased sensitivity to treatment. Compounds with more than 20% missing AUC values were excluded. Next, K-nearest neighbor imputation was applied to impute the missing AUC values of the remaining compounds.We then used the pRRophetic package (Geeleher et al., 2014) to construct a ridge regression model between the CCLs expression profiles and drug sensitivity AUC values, and subsequently performed drug sensitivity prediction on the TPM data from TCGA. We subsequently retrieved the target information of potential sensitive drugs from DrugBank. Using the TCGA-LIHC count data, we analyzed the differential expression of these targets between tumor and normal tissues, as well as between high risk and low risk groups.

## 3 Results

### 3.1 Multiomics consensus prognosis-related molecular subtypes of HCC

**FIGURE 1:**
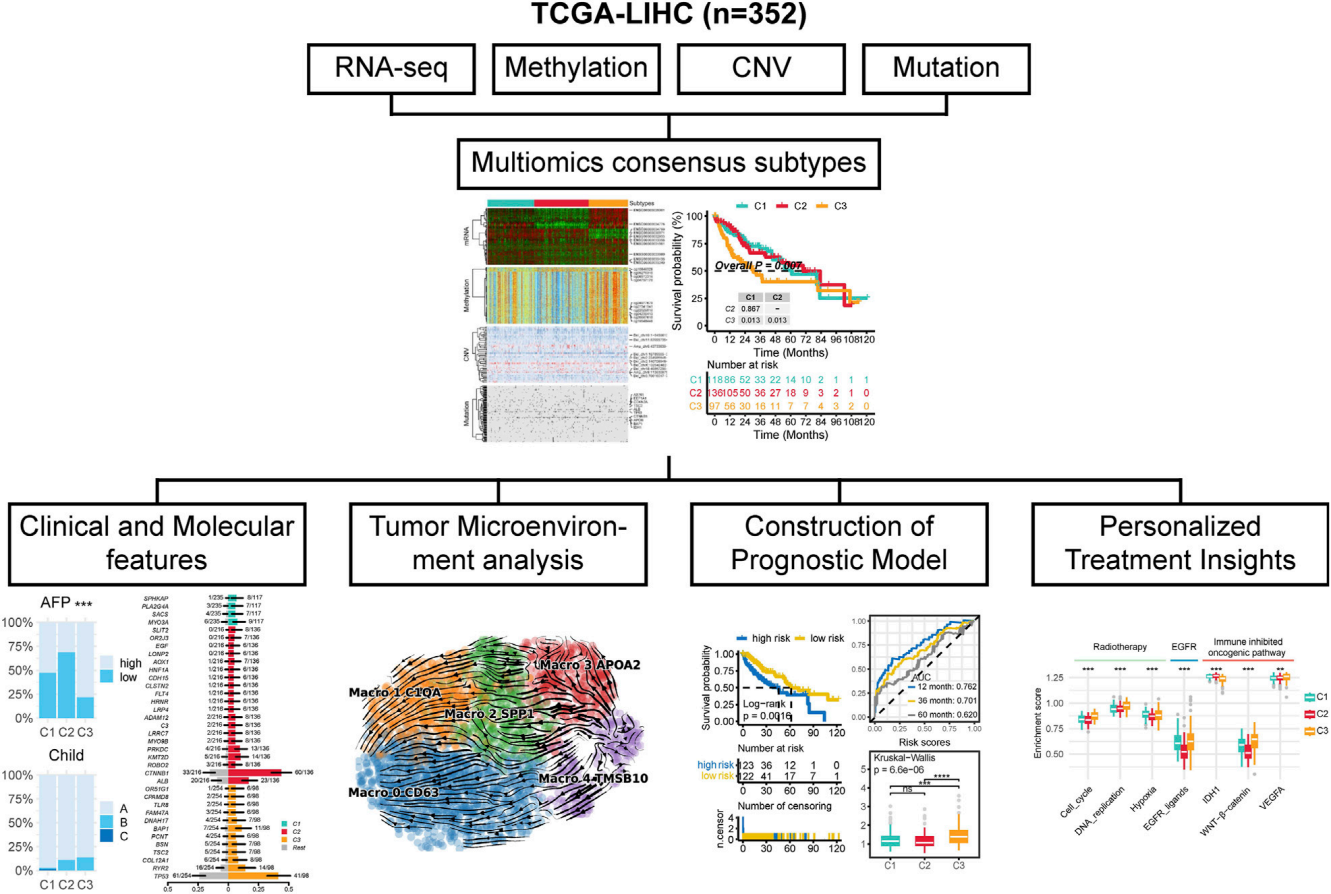
The workflow of this study.

**FIGURE 2:**
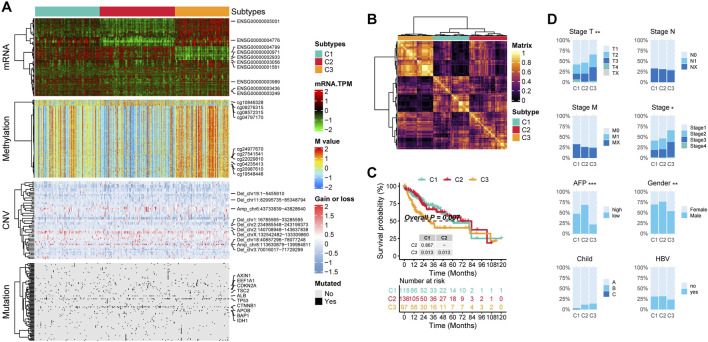
The multi-omics integrative subtypes of TCGA-LIHC. **(A)** Comprehensive heatmap of consensus ensemble subtypes, incorporating mRNA expression, DNA methylation, CNV, and mutations. **(B)** Consensus clustering matrix for the three novel prognostic subtypes based on 10 clustering algorithms. **(C)** Kaplan-Meier survival analysis of the consensus ensemble subtypes. **(D)** Association between the three subtypes and clinical characteristics.

The workflow of this research is shown in Figure 1. In this study, by integrating multi-omics data, we selected a total of 352 patients for further analysis (Supplementary Table S1). We try to identify distinct multi-omics molecular subtypes (molecular expression data, encompassing mRNA, methylation, CNV, and somatic mutations) of HCC by integrating the results of ten different multi-omics clustering algorithms. To determine the optimal number of clusters, we evaluated the clustering stability and structural quality across a range of cluster numbers (k = 2–8). The getClustNum function from the MOVICS package suggested that k = 2 was the optimal choice, supported by a high CPI, indicating excellent clustering stability (Supplementary Figure S3A). However, the corresponding Gap statistics was relatively low, suggesting limited discriminatory power. To address this, we explored solutions with higher cluster numbers. In the three cluster solution, Cluster one exhibited expression patterns in both the transcriptome and methylation profiles that were similar to, yet distinct from those of the other two clusters (Figure 2A). This suggests that Cluster 1 may represent a “transitional” state with potential biological relevance. Notably, Silhouette analysis further supported the three cluster model: while Cluster two exhibited relatively low silhouette width, which may be attributed to internal heterogeneity, Clusters one and three showed moderate to high silhouette values, indicating that these groups are well separated and internally coherent (Supplementary Figure S3B). The average silhouette width across all samples also indicated a reasonable clustering structure. Although increasing the number of clusters could yield finer stratifications, it would also substantially increase the complexity of downstream analyses, including functional annotation, biological validation, and clinical interpretation. Moreover, both the CPI and Gap statistics showed no substantial improvement beyond three clusters (Supplementary Figure S3A). Therefore, we selected the three cluster solution as it provides a balanced trade off between biological separability, clustering stability, and analytical feasibility for subsequent investigations. By analyzing the results of consensus clustering, we conclude that our classification outcomes are robust and represent a consensus across multiple algorithms (Figure 2B). Survival analysis revealed significantly different prognoses among the molecular subtypes, with patients in the C3 subtype exhibiting the worst prognosis (p = 0.007; Figure 2C). Patients in the C3 subtype also exhibited a strong association with higher Stage T, more advanced overall stage, higher AFP levels, and a higher prevalence in females (Figure 2D).

### 3.2 Molecular characteristics of multi-omics subtypes

**FIGURE 3:**
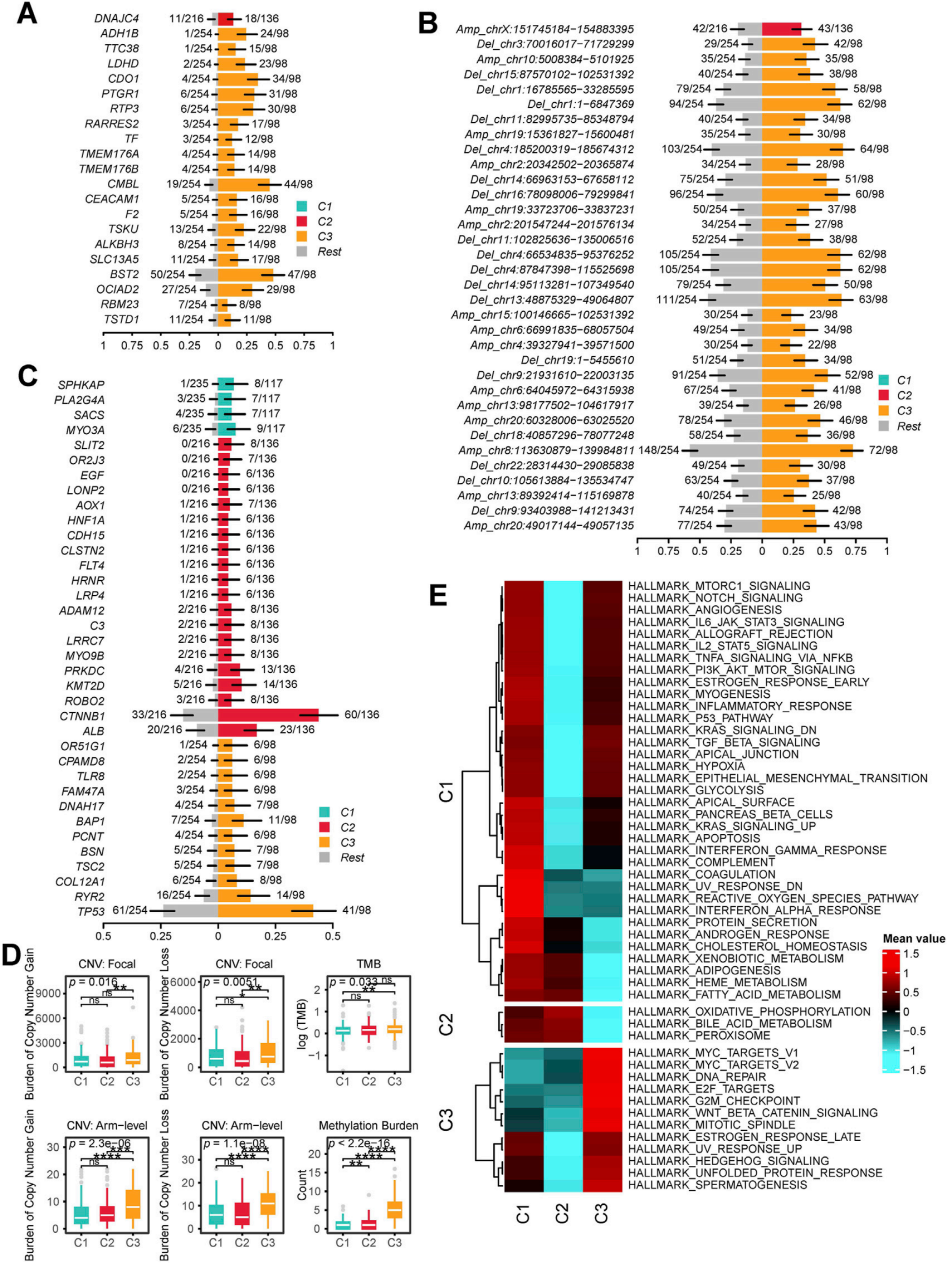
Molecular Characteristics of Multi-Omics Integrative Subtypes. **(A)** subtype specific methylation silenced genes: each row represents a gene that is methylation silenced in a specific subgroup. **(B)** subtype specific CNV Regions: Each row represents a chromosomal region with significant copy number alterations. The y-axis indicates whether the region underwent amplification or deletion, along with the chromosome number and coordinates. **(C)** subtype specific Mutations: Each row represents a mutated gene. In **(A)**, **(B)** and **(C)**, the x-axis represents the proportion of samples exhibiting the alteration. The right side colored sections indicate the proportion of methylation silenced genes, CNV alterations or mutations occurring in the given subtype, while the left side gray section represents the proportion in patients outside this subtype. **(D)** Genomic and Epigenomic Alteration Burden: The first row shows the burden of focal CNVs in each subtype, the second row represents the burden of arm level CNVs, and the third row displays the mutation burden and methylation silencing burden for each subtype. **(E)** subtype specific Significantly Upregulated HALLMARK Pathways: Each row in the heatmap represents the average level of a specific HALLMARK pathway within a subtype, with red indicating high levels and blue indicating low levels. (ns, not significant; *, P ≤ 0.05; **, P ≤ 0.01; ***, P ≤ 0.001; ****, P ≤ 0.0001).

First, by comparing the proportion of methylation silencing and CNV, we found that the vast majority of methylation silenced genes and CNV were significantly enriched in C3 patients, whereas no subtype specific methylation silencing or CNV were observed in C1 patients (Figures 3A, B). Next, we analyzed the subtype specific mutations. C1 patients exhibited fewer subtype specific mutations with relatively low frequencies. However, an interesting pattern emerged: C2 patients had a significantly higher frequency of CTNNB1 mutations, while C3 patients exhibited significantly more TP53 mutations (Figure 3C).

We then calculated the copy number burden, mutation burden, and methylation silencing burden for each subtype (Figure 3D). The results showed that C3 patients had significantly elevated focal and arm level CNV burdens, mutation burdens, and methylation silencing burdens (P < 0.05).

Finally, we performed ssGSEA to obtain HALLMARK scores for all patients. The results indicated that C1 patients exhibited the highest levels of HALLMARK_COAGULATION, HALLMARK_UV_RESPONSE_DN, HALLMARK_REACTIVE_OXYGEN_SPECIES_PATHWAY, and HALLMARK_INTERFERON_ALPHA_RESPONSE. In C2 patients, proliferation and immunity related pathways were downregulated, while metabolism related pathways (e.g., HALLMARK_OXIDATIVE_PHOSPHORYLATION, HALLMARK_BILE_ACID_METABOLISM, HALLMARK_PEROXISOME) were significantly upregulated. In C3 patients, both proliferation related (HALLMARK_MYC_TARGETS_V1, HALLMARK_MYC_TARGETS_V2, HALLMARK_E2F_TARGETS) and immunity related pathways (e.g., HALLMARK_NOTCH_SIGNALING, HALLMARK_IL6_JAK_STAT3_SIGNALING, HALLMARK_ALLOGRAFT_REJECTION) were highly activated, whereas metabolism related pathways were downregulated (Figure 3E).

### 3.3 TME characteristics of multi-omics subtypes

**FIGURE 4:**
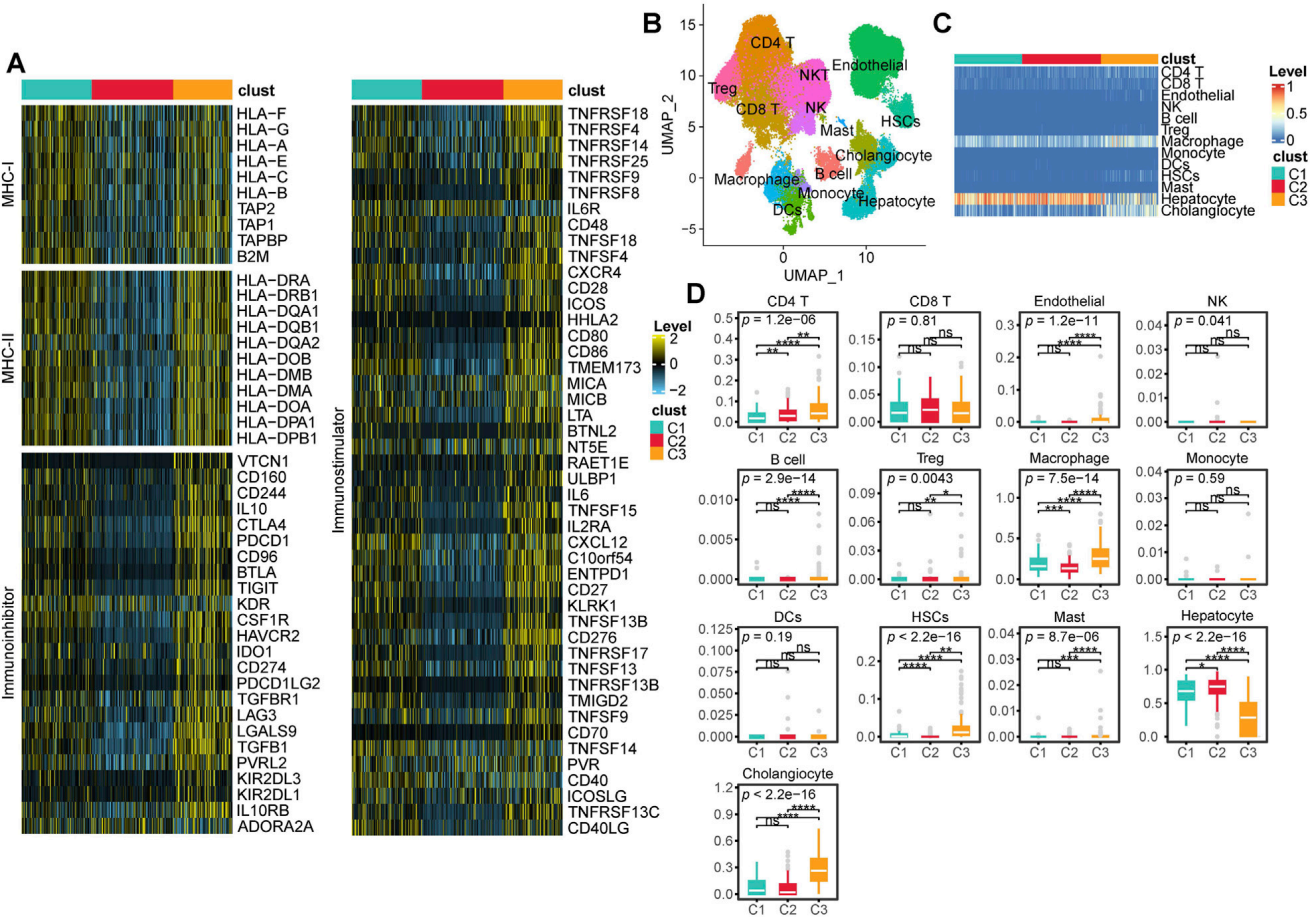
TME characteristics across multi-Omics Subtypes. **(A)** Heatmap showing the expression levels of MHC-I, MHC-II, immunoinhibitor, and immunostimulator across different subtypes. **(B)** UMAP visualization of cell annotation results from the GSE156337 dataset. **(C)** Heatmap displaying the deconvolution results of CIBERSORT based cell annotations from GSE156337 applied to the TCGA-LIHC cohort, where red indicates a higher proportion of a given cell type and blue indicates a lower proportion. **(D)** Box plots illustrating the deconvolution results from CIBERSORT along with statistical analysis. (ns, not significant; *, P ≤ 0.05; **, P ≤ 0.01; ***, P ≤ 0.001; ****, P ≤ 0.0001).

First, we compared the expression levels of MHC-I, MHC-II, immunoinhibitory molecules, and immunostimulatory molecules across different subtypes. We found that these molecules exhibited the highest expression levels in C3 patients, followed by slightly lower levels in C1 patients, and the lowest expression levels in C2 patients (Figure 4A). To further investigate the differences in the TME among different subtypes, we focused on the cellular level variations across subtypes. We first performed batch effect correction and cell type annotation on the human HCC single-cell dataset GSE156337 (Figure 4B). The correspondence between each cell type and its markers is shown in Supplementary Figure S4. We used the FindAllMarkers function in Seurat to identify marker genes for all cell subpopulations and applied them to CIBERSORTx for deconvolution in the TCGA-LIHC dataset. (Supplementary Table S2). Our analysis revealed that the predominant cell types across all patients were macrophages, hepatocytes, and cholangiocytes (Figure 4C; Supplementary Table S3). Notably, compared to C1 and C2 patients, C3 patients exhibited a significantly lower proportion of hepatocytes and a significantly higher proportion of cholangiocytes and macrophages (Figures 4C,D). Additionally, CD4 T cells, B cells, regulatory T (Treg) cells, HSCs, and mast cells were significantly enriched in C3 patients. However, no significant differences in the proportion of CD8 T cells were observed among the subtypes (Figure 4D).

### 3.4 Diversity and dynamics of macrophages in the HCC TME

**FIGURE 5:**
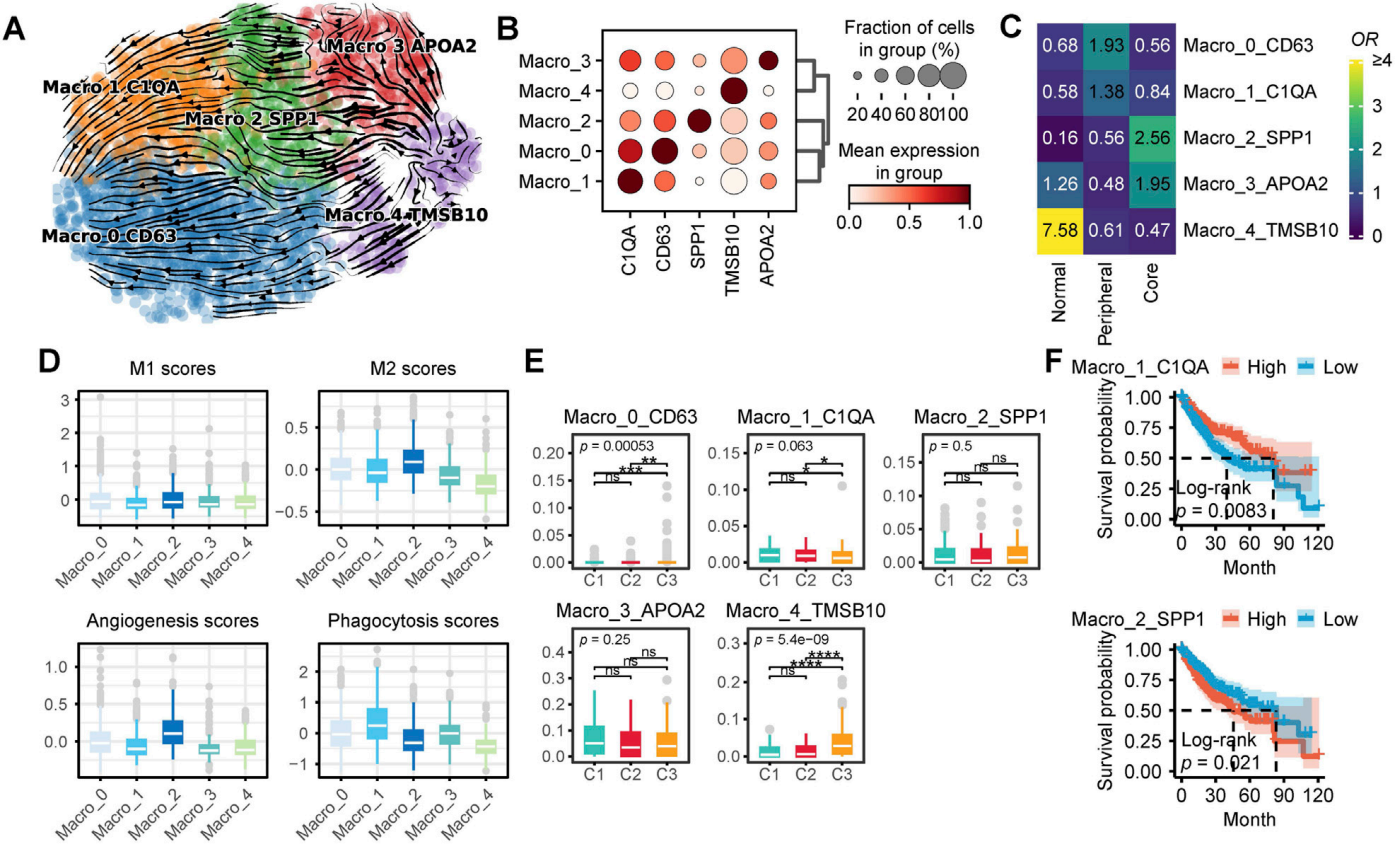
Characteristics of Macrophages in HCC. **(A)** Macrophage subpopulations and RNA velocity trajectory. **(B)** Marker genes of different macrophage subpopulations. **(C)** Enrichment of different macrophage subtypes across various tissue types. A higher odds ratio (OR) and more yellow coloration indicate greater enrichment of a specific macrophage subtype in the corresponding tissue type, whereas a more blue coloration suggests the opposite. **(D)** Functional scores of macrophages for M1 and M2 polarization, angiogenesis, and phagocytosis. **(E)** Proportions of different macrophage subpopulations across distinct patient subtypes. **(F)** Survival analysis of single-cell CIBERSORTx deconvolution in TCGA-LIHC. (ns, not significant; *, P ≤ 0.05; **, P ≤ 0.01; ***, P ≤ 0.001; ****, P ≤ 0.0001).

To delineate the heterogeneity of macrophages in HCC, we extracted 2,615 macrophages from the single-cell dataset and performed reclustering, ultimately identifying five distinct macrophage subpopulations. Based on classical macrophage markers and differentially expressed genes among the subpopulations, we designated these clusters as Macro_0_CD63, Macro_1_C1QA, Macro_2_SPP1, Macro_3_APOA2, and Macro_4_TMSB10 (resolution = 0.4) (Figures 5A, B). RNA velocity analysis suggested that Macro_4_TMSB10 serves as the differentiation origin of all macrophages, with two major differentiation trajectories. In the first trajectory, Macro_4_TMSB10 gradually evolves into Macro_3_APOA2, then transitions into Macro_2_SPP1, and ultimately differentiates into Macro_1_C1QA. The second trajectory involves the differentiation of Macro_4_TMSB10 into Macro_0_CD63 (Figure 5A).

The proportions of different macrophage subpopulations varied across peritumoral tissue, the periphery of HCC, and the tumor core (Figure 5C). Macro_4_TMSB10 was predominantly enriched in peritumoral tissue, while Macro_3_APOA2 was enriched in both peritumoral tissue and the tumor core. In contrast, Macro_2_SPP1 was exclusively enriched in the tumor core, whereas Macro_0_CD63 and Macro_1_C1QA were primarily enriched in the tumor periphery. Functionally, Macro_2_SPP1 exhibited the highest M2 polarization and angiogenesis scores, whereas Macro_0_CD63 and Macro_1_C1QA displayed relatively higher phagocytic activity scores (Figure 5D). To further investigate the distribution of these macrophage subpopulations in the TCGA-LIHC cohort, we analyzed CIBERSORTx results. Notably, C1 patients exhibited a significantly higher proportion of Macro_2_SPP1, whereas C3 patients had significantly higher proportions of Macro_0_CD63, Macro_2_SPP1, and Macro_4_TMSB10 (Figure 5E). Survival analysis indicates that patients with high levels of Macro_1_C1QA have a significantly better prognosis, whereas those with high levels of Macro_2_SPP1 exhibit the opposite trend. (Figure 5F).

### 3.5 Diversity of hepatocyte heterogeneity in the HCC TME

**FIGURE 6:**
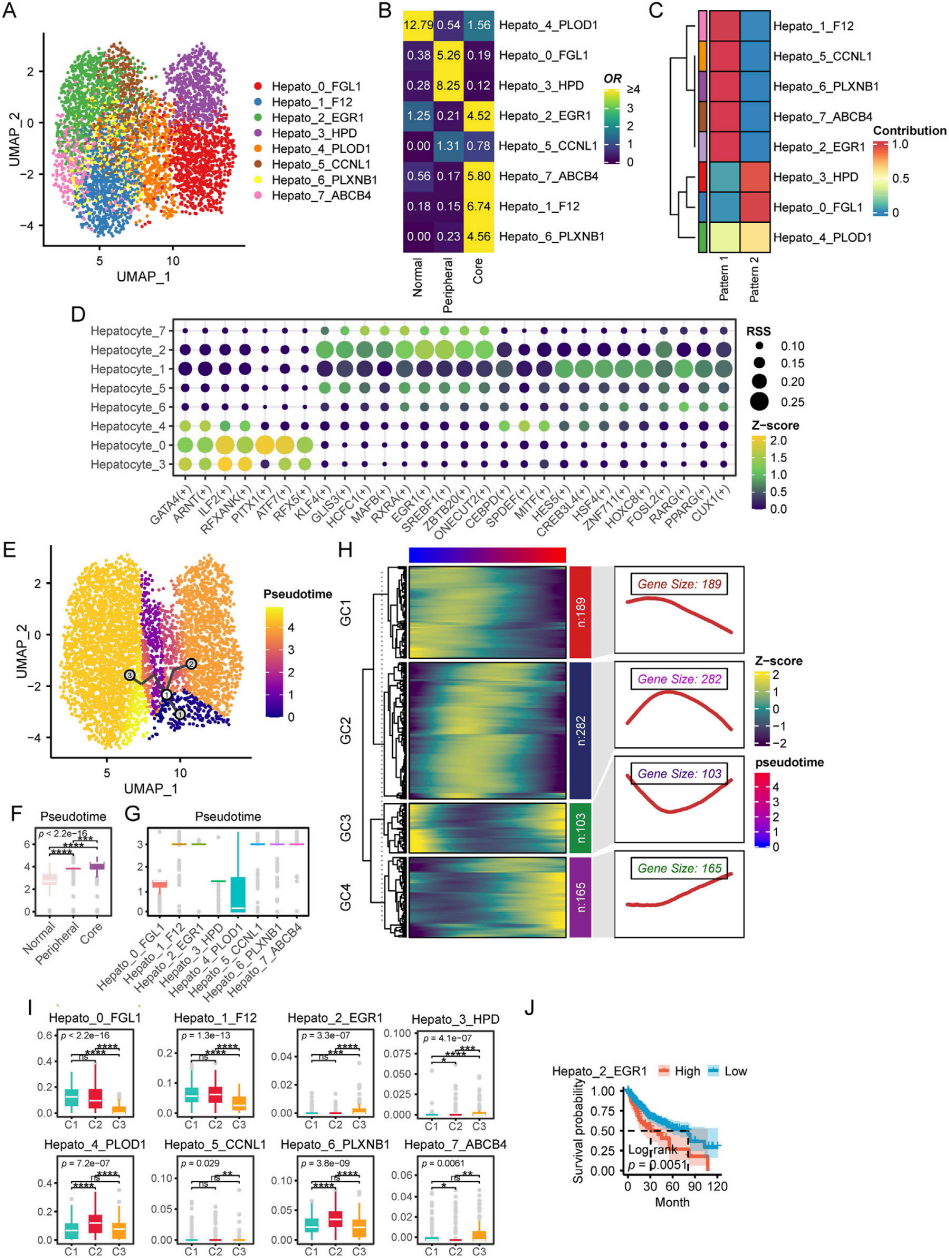
Characteristics of hepatocytes in HCC. **(A)** UMAP plot of hepatocytes subpopulations. **(B)** Enrichment of different hepatocytes subtypes across various tissue types. A higher OR and more yellow coloration indicate greater enrichment of a specific macrophage subtype in the corresponding tissue type, whereas a more blue coloration suggests the opposite. **(C)** NMF analysis of SCENIC transcription factor expression patterns across different cellular subpopulations. Each row represents a cellular subpopulation, and each column corresponds to a transcription factor expression pattern identified by NMF. A redder color indicates a stronger tendency of the transcription factors in that subpopulation to adopt the corresponding expression pattern, whereas a bluer color indicates the opposite tendency. **(D)** This dot plot illustrates the regulatory activity of TFs across different hepatocyte subpopulations identified by pySCENIC. Each row represents a hepatocyte subpopulation, while each column corresponds to a specific TFs. The size of each dot indicates the RSS, reflecting the specificity of a transcription factor in a given subpopulation. Larger dots represent higher RSS values, signifying stronger regulatory specificity. The color gradient represents the Z score of transcription factor activity, which measures the relative expression level of each TF. Yellow green indicates stronger TF activity. Darker colors indicate weaker TF activity. **(E)** Pseudotime Trajectory Analysis with Monocle3. This UMAP plot visualizes the pseudotime trajectory of cells inferred by Monocle3. Cells are colored by pseudotime, from purple (early state) to yellow (late state).The black line represents the inferred differentiation trajectory. Circular nodes indicate key branching points. **(F)** Pseudotime levels across different tissue types. **(G)** Pseudotime of different cellular subpopulations. **(H)** Pseudotime ordered gene expression patterns. This heatmap illustrates the dynamic expression of genes across pseudotime, clustered into four modules (GC1–GC4) based on similar expression trends. The heatmap colors represent Z score normalized expression levels, with blue indicating low expression and yellow indicating high expression. The right panels display smoothed expression curves along pseudotime for each module, highlighting distinct transcriptional dynamics. The gene size denotes the number of genes in each module, revealing key patterns of gene regulation during cellular transitions.**(I)** Single-cell CIBERSORTx deconvolution results of TCGA-LIHC. **(J)** Survival analysis of single-cell CIBERSORTx deconvolution in TCGA-LIHC. (ns, not significant; *, P ≤ 0.05; **, P ≤ 0.01; ***, P ≤ 0.001; ****, P ≤ 0.0001).

In this section, we focus on the heterogeneity and functionality of hepatocytes within the HCC TME. We extracted a total of 4,653 hepatocytes from scRNA data for further analysis. We first identified eight distinct hepatocyte subpopulations (resolution = 0.8) along with their corresponding marker genes (Figure 6A; Supplementary Figure S5A). Notably, these hepatocyte subpopulations exhibited distinct spatial distribution preferences within the tissue. Specifically, Hepato_4_PLOD1 was predominantly localized in normal tissue, whereas Hepato_0_FGL1, Hepato_3_HPD, and Hepato_5_CCNL1 were enriched in the tumor periphery. In contrast, Hepato_2_EGR1, Hepato_7_ABCB4, Hepato_1_F12, and Hepato_6_PLXNB1 were preferentially distributed in the tumor core (Figure 6B). By performing HALLMARK scoring on single cells from each subpopulation, we found that Hepato_0_FGL1 and Hepato_3_HPD exhibited the highest levels of REACTIVE_OXYGEN_SPECIES_PATHWAY, OXIDATIVE_PHOSPHORYLATION, DNA_REPAIR, and MYC_TARGETS_V1. Among them, Hepato_0_FGL1 displayed the highest levels of COAGULATION, ANGIOGENESIS, and COMPLEMENT. In contrast, cell subpopulations predominantly located in the tumor core region exhibited elevated levels of TNFA_SIGNALING_VIA_NFKB and BILE_ACID_METABOLISM (Supplementary Figure S5B).

Next, we applied pySCENIC to identify potential TFs associated with each hepatocyte subpopulation. We further employed NMF to analyze the Regulon Specificity Score (RSS) profiles generated by pySCENIC. Our findings revealed that hepatocytes located in the tumor periphery exhibited a transcriptional regulatory pattern distinct from those within the tumor core (Figure 6C). Specifically, hepatocytes at the tumor periphery predominantly expressed TFs such as ILF2, ATF7, and RFXANK, while those in the tumor core were enriched for KLF4, EGR1, and HES5 (Figure 6D).

To further investigate the evolutionary trajectory of hepatocytes in HCC, we performed pseudotime trajectory analysis using Monocle3. The inferred trajectory suggested that hepatocytes originate from normal liver tissue and subsequently diverge into two major branches: one leading to tumor periphery associated hepatocytes, and the other giving rise to tumor core associated hepatocytes (Figure 6E). Importantly, hepatocytes located in the tumor periphery appeared to be at earlier pseudotime compared to those within the tumor core (Figures 6F, G), suggesting a progressive evolution of hepatocyte states during tumor development. Subsequently, we identified genes that exhibited significant associations with pseudotime progression, revealing four distinct expression clusters (GC1–GC4) (Figure 6H). GC1 genes were predominantly highly expressed at the early stages of pseudotime, whereas GC2 genes were primarily enriched at the midpoint of the trajectory. In contrast, GC3 genes displayed high expression levels at both the beginning and the end of pseudotime, while GC4 genes were specifically upregulated at the terminal stages of pseudotime. KEGG enrichment analysis revealed distinct functional signatures for each pseudotime gene cluster. GC1 genes were significantly enriched in proliferation and immunity related pathways, including hsa04151: PI3K-Akt signaling pathway and hsa04060: Cytokine-cytokine receptor interaction. GC2 genes were enriched in a broader range of pathways associated with tumorigenesis, immunity, and signal transduction. In contrast, GC3 genes were exclusively enriched in hsa00830: Retinol metabolism and hsa04360: Axon guidance, suggesting their involvement in metabolic and developmental processes. GC4 genes were predominantly associated with immune and extracellular matrix related pathways, such as hsa04514: Cell adhesion molecules, hsa05332: Graft versus host disease, and hsa04145: Phagosome. Notably, GC4 genes were also significantly enriched in hsa04310: Wnt signaling pathway, indicating a potential role in cellular differentiation and tissue remodeling (Supplementary Figure S5C).

Finally, based on the results of CIBERSORTx, we compared the distribution proportions of different cellular subpopulations among patients with different multi-omics subtypes. The results showed that Hepato_0_FGL1 and Hepato_1_F12 were significantly enriched in patients from the C1 and C2 groups. Additionally, Hepato_4_PLOD1 and Hepato_6_PLXNB1 exhibited significantly higher proportions in C2 patients, while Hepato_2_EGR1 and Hepato_3_HPD were significantly more abundant in C3 patients (Figure 6I). Survival analysis indicated that a higher level of Hepato_2_EGR1 was significantly associated with poorer prognosis (Figure 6J).

### 3.6 Diversity of cholangiocyte heterogeneity in the HCC TME

**FIGURE 7:**
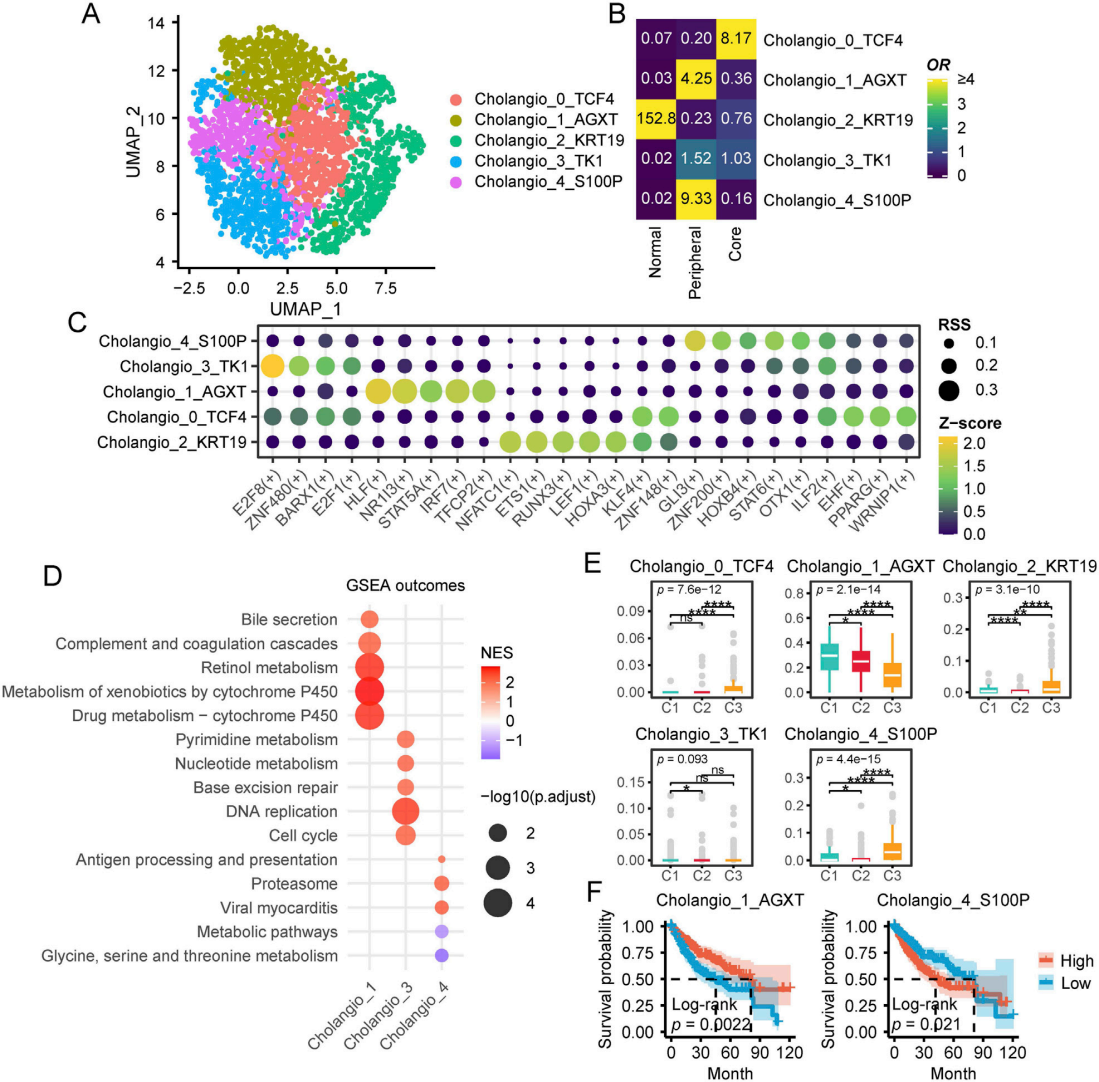
Characteristics of cholangiocytes in HCC. **(A)** UMAP plot of cholangiocytes subpopulations. **(B)** Enrichment of different cholangiocytes subtypes across various tissue types. A higher OR and more yellow coloration indicate greater enrichment of a specific macrophage subtype in the corresponding tissue type, whereas a more blue coloration suggests the opposite. **(C)** This dot plot illustrates the regulatory activity of TFs across different hepatocyte subpopulations identified by pySCENIC. Each row represents a hepatocyte subpopulation, while each column corresponds to a specific TFs. The size of each dot indicates the RSS, reflecting the specificity of a transcription factor in a given subpopulation. Larger dots represent higher RSS values, signifying stronger regulatory specificity. The color gradient represents the Z score of transcription factor activity, which measures the relative expression level of each TF. Yellow green indicates stronger TF activity. Darker colors indicate weaker TF activity. **(D)** GSEA Enrichment Analysis of Cholangiocyte Subpopulations. The x-axis represents cholangiocyte subpopulations (Cholangio_1, Cholangio_3, Cholangio_4), while the y-axis lists enriched KEGG pathways. Bubble size corresponds to -log10 (p.adjust), indicating statistical significance, and bubble color represents the Normalized Enrichment Score (NES), with red indicating positive enrichment and purple indicating negative enrichment. **(E)** Single-cell CIBERSORTx deconvolution results of TCGA-LIHC. **(F)** Survival analysis of single-cell CIBERSORTx deconvolution in TCGA-LIHC. (ns, not significant; *, P ≤ 0.05; **, P ≤ 0.01; ***, P ≤ 0.001; ****, P ≤ 0.0001).

Previous CIBERSORTx results indicated that C3 patients exhibited higher levels of cholangiocytes, which piqued our interest. Therefore, we conducted a more in depth analysis of cholangiocytes in this section. First, we extracted 2,468 cholangiocytes from the single-cell dataset and classified them into five subpopulations using resolution = 0.8. Each subpopulation was named based on its marker genes (Figure 7A; Supplementary Figure S6). Tissue distribution preference analysis revealed that Cholangio_2_KRT19 was predominantly located in peritumoral tissues, Cholangio_1_AGXT and Cholangio_4_S100P were mainly found in the tumor periphery, Cholangio_0_TCF4 was concentrated in the tumor core, while Cholangio_3_TK1 was distributed across both the core and periphery (Figure 7B).

TFs analysis of cholangiocyte subpopulations revealed distinct regulatory patterns across different subgroups (Figure 7C). Specifically, Cholangio_4_S100P exhibited high expression of GLI3, ZNF200, and HOXB4, while Cholangio_3_TK1 specifically expressed E2F8, ZNF480, and E2F1. In Cholangio_1_AGXT, HLF, NR1I3, and IRF7 were significantly enriched, whereas ILF2 and EHF were highly expressed in Cholangio_0_TCF4. Additionally, Cholangio_2_KRT19 showed strong expression of NFATC1, ETS1, and LEF1.

To further investigate the functional characteristics of different cholangiocyte subpopulations, we identified subpopulation specific markers using Seurat and performed GSEA based KEGG pathway enrichment analysis (Figure 7D). Notably, Cholangio_0_TCF4 and Cholangio_2_KRT19 did not exhibit significant pathway enrichment. However, Cholangio_1_AGXT was strongly associated with metabolism and complement related pathways, while Cholangio_3_TK1 was highly enriched in DNA synthesis, repair, and cell cycle pathways. In contrast, Cholangio_4_S100P was significantly linked to proteasome and immune related pathways.

CIBERSORTx analysis further examined the distribution of cholangiocyte subpopulations across patient groups (Figure 7E). Cholangio_0_TCF4, Cholangio_2_KRT19, and Cholangio_4_S100P were most abundant in C3 patients, whereas Cholangio_1_AGXT was significantly enriched in C1 patients. Survival analysis revealed that higher levels of Cholangio_1_AGXT were associated with better prognosis, whereas higher levels of Cholangio_4_S100P correlated with poorer prognosis (Figure 7F).

### 3.7 Cell-cell communication analysis

**FIGURE 8:**
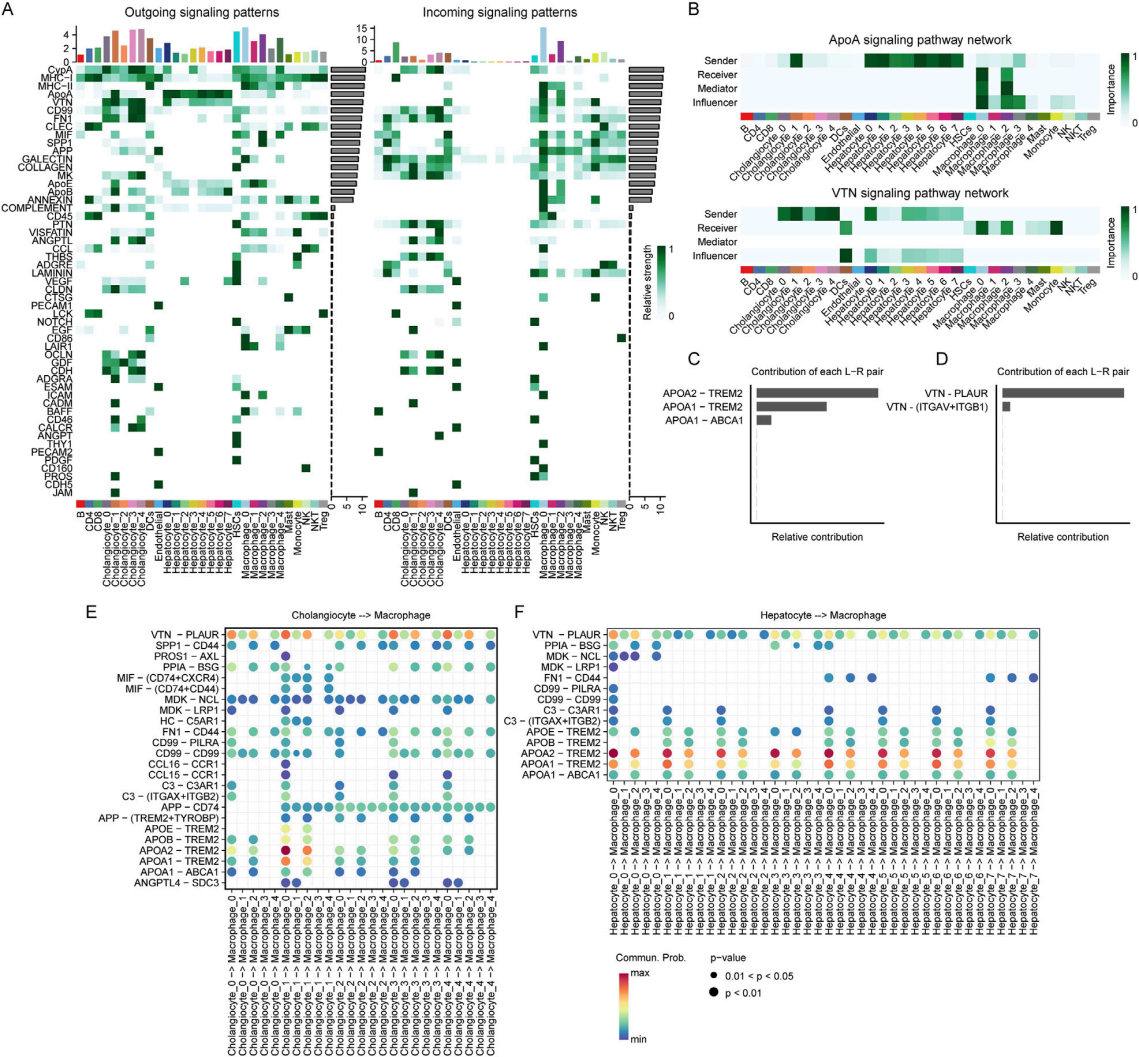
Cell communications among hepatocytes, cholangiocytes and macrophages. **(A)** Outgoing and incoming signaling patterns of ligand-receptor interactions across different cell subpopulations. The heatmap represents the relative strength of signaling interactions, with darker green indicating higher interaction strength. Bar plots at the top indicate the number of interactions for each subpopulation. Cell types are annotated at the bottom. **(B)** Comparison of the ApoA and VTN signaling pathway networks across different cell subpopulations. The heatmaps represent the importance of each cell type as a sender, receiver, mediator, or influencer in the signaling network. Darker green indicates higher importance. Cell types are annotated at the bottom. **(C, D)** Relative contributions of ligand-receptor (L–R) pairs in ApoA **(C)** and VTN **(D)** signaling pathways. Bar lengths represent the relative contribution of each L-R interaction. ApoA-TREM2 and VTN-PLAUR exhibit the highest contributions in their respective pathways. **(E)** Ligand-receptor interactions between cholangiocyte and macrophage subpopulations. Each dot represents an interaction, with size and color indicating interaction strength. The x-axis denotes cholangiocyte to macrophage signaling, while the y-axis lists specific ligand-receptor pairs. **(F)** Ligand-receptor interactions between hepatocyte and macrophage subpopulations. Each dot represents an interaction, with size and color indicating interaction strength. The x-axis denotes cholangiocyte to macrophage signaling, while the y-axis lists specific ligand-receptor pairs.

To further investigate the TME of HCC, we conducted cell-cell communication analysis using CellChat. First, we analyzed the number of incoming and outgoing signaling interactions in each cell cluster. Our results showed that in terms of outgoing signals, hepatocytes were primarily involved in the ApoA signaling pathway, while cholangiocytes exhibited higher levels of VTN, CypA, and MHC-I signaling pathways. Macrophages displayed elevated activity in both MHC-I and MHC-II pathways (Figure 8A). Regarding incoming signals, hepatocytes exhibited minimal incoming signaling activity. In contrast, cholangiocytes showed high levels of CypA, GALECTIN, and COLLAGEN signaling pathways. Notably, among macrophage subsets, Macrophage_0 and Macrophage_2 displayed highly similar incoming signaling patterns, with both exhibiting elevated levels of MHC-II, ApoA, VTN, SPP1, and APP signaling pathways.

We found that the majority of ApoA signals originating from hepatocytes and VTN signals from cholangiocytes were primarily received by Macrophage_0_CD63 and Macrophage_2_SPP1 (Figure 8B). To further investigate the contribution of different ligand-receptor pairs within these signaling pathways, we analyzed their relative contributions. Our results revealed that in the ApoA pathway, APOA1–TREM2 and APOA2–TREM2 were the two ligand-receptor pairs with the highest contributions (Figure 8C). In the VTN pathway, VTN–PLAUR exhibited the highest contribution (Figure 8D).

To further elucidate the role of VTN-PLAUR, we analyzed the ligand-receptor interactions between cholangiocyte subpopulations and macrophage subpopulations. Our findings revealed significantly elevated interaction levels from Cholangio_0_TCF4, Cholangio_1_AGXT, Cholangio_3_TK1 and Cholangio_4_S100P to Macro_0_CD63 and Macro_2_SPP1 (Figure 8E). Regarding APOA1-TREM2 and APOA2-TREM2, we examined the ligand-receptor interactions between hepatocyte subpopulations and macrophage subpopulations. The results demonstrated that all hepatocyte subpopulations exhibited significantly high levels of interaction with Macro_0_CD63 and Macro_2_SPP1 (Figure 8F).

### 3.8 Construction the prognostic score system

**FIGURE 9:**
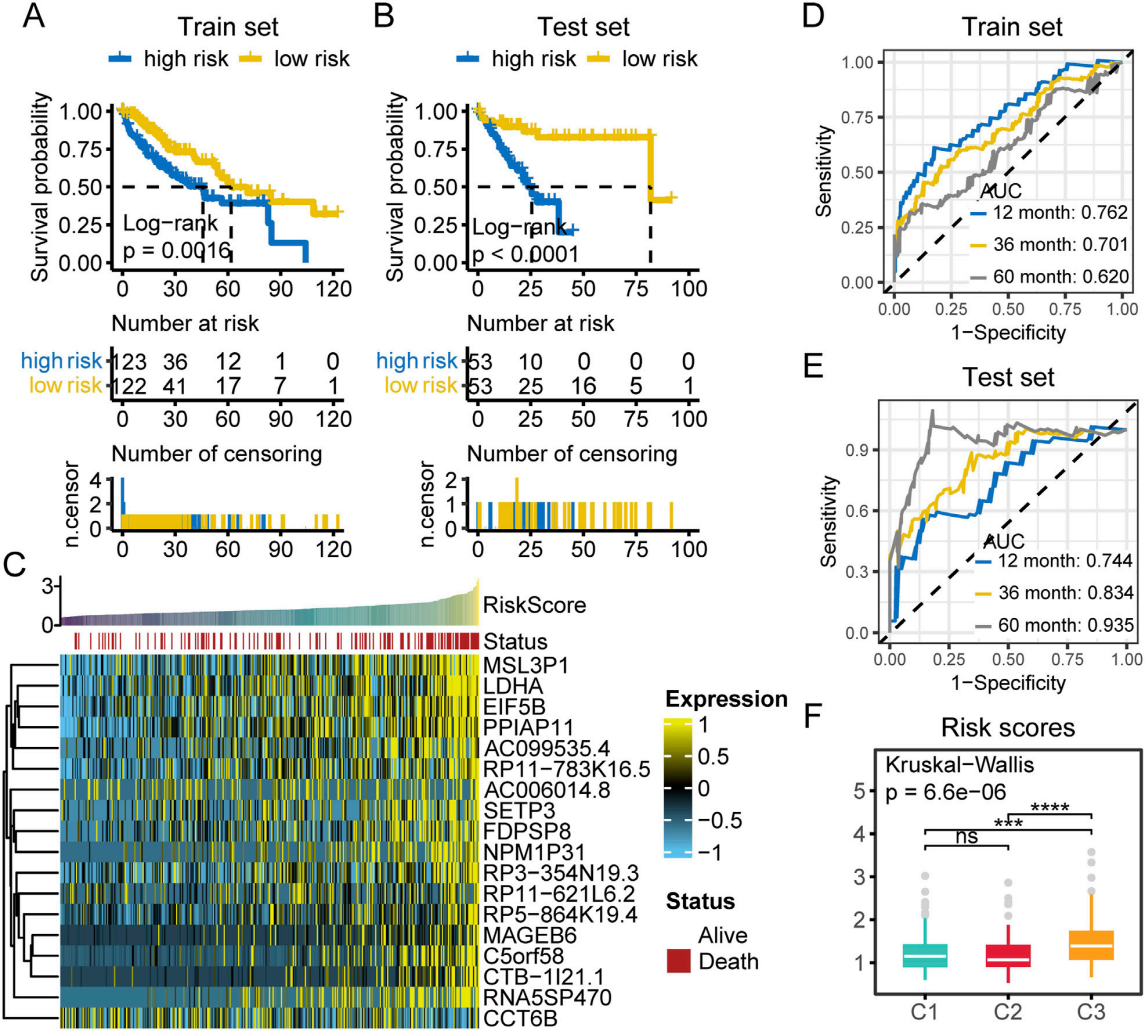
Prognostic risk assessment in HCC patients. **(A)** Survival analysis of high and low risk patients in the training set. **(B)** Survival analysis of high and low risk patients in the test set. **(C)** Heatmap of the expression of 18 LASSO selected prognostic genes in TCGA-LIHC samples. Samples are ordered by increasing risk score, with survival status annotated above (red: death, white: alive). Gene expression is scaled and color coded (yellow: high, blue: low), with most genes highly expressed in high risk cases. **(D)** The time dependent ROC curves of prognostic classifier of train set. AUCs at 1, 3, and 5 years were used to assess prognostic accuracy. **(E)** The time dependent ROC curves of prognostic classifier of test set. AUCs at 1, 3, and 5 years were used to assess prognostic accuracy. **(F)** Comparison of risk scores among the three multi-omics subtypes. (ns, not significant; *, P ≤ 0.05; **, P ≤ 0.01; ***, P ≤ 0.001; ****, P ≤ 0.0001; AUC, area under the curve; n.censor, number censored).

To more accurately assess the prognosis of patients with different subtypes and enhance its applicability in clinical practice, we developed a prognostic scoring system. First, we performed univariate Cox analysis to identify genes significantly associated with prognosis (P < 0.01), resulting in the selection of 7,187 prognostically significant genes. Subsequently, we applied the LASSO method to calculate the prognostic risk score based on the expression profiles and survival data of these survival associated genes. Ultimately, the LASSO method identified 18 survival associated genes with the strongest predictive power. Detailed information on these 18 survival associated genes and their regression coefficients is provided in Supplementary Table S4. The prognostic risk score was calculated by integrating the expression levels of the LASSO selected signature genes with their corresponding LASSO regression coefficients. Patients were then stratified into high risk and low risk groups based on the median prognostic score. Kaplan-Meier survival analysis demonstrated that patients in the high risk group had significantly worse survival outcomes than those in the low risk group in both the training and test sets (Figures 9A,B). We found that most of the deceased cases were concentrated in the high risk group (Figure 9C). Moreover, the majority of the 18 LASSO derived markers were highly expressed in the high risk population (Figure 9C). Additionally, we performed receiver operating characteristic (ROC) analysis on the prognostic risk score, which indicated that our prognostic scoring model exhibited excellent predictive performance for both short term and long term survival (Figures 9D, E). We compared the survival risk among patients with different subtypes and found that the C3 group had the highest survival risk (Figure 9F). Univariate and multivariate Cox regression analyses further confirmed that the prognostic risk score was an independent prognostic predictor in the TCGA-LIHC cohort (Supplementary Tables S5, 6).

### 3.9 Assessing the treatment sensitivity of patients in different groups

**FIGURE 10:**
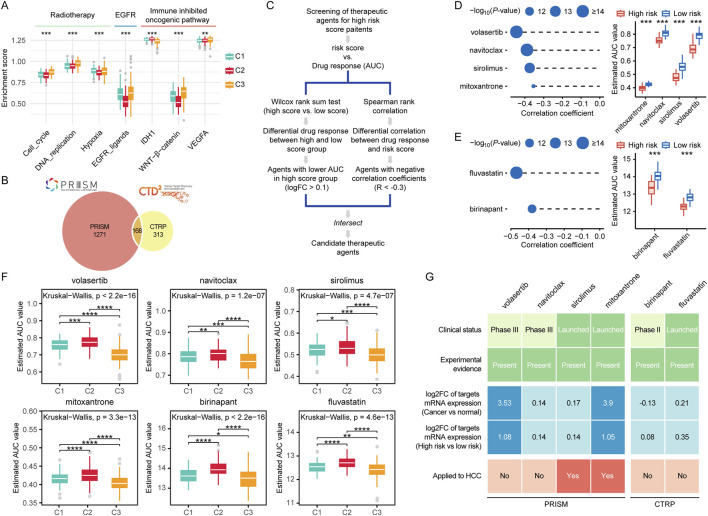
Prognostic risk scores accurately predicted therapeutic opportunities. **(A)** Differences in therapeutic signatures related to radiotherapy, EGFR signaling, and immune inhibited oncogenic pathways among different molecular subtypes. **(B)** A Venn diagram of compounds from the CTRP and PRISM datasets. **(C)** The workflow of identifying potential therapeutic compounds. **(D, E)** The left panel shows the Spearman correlation between the prognostic risk scores and compounds responses predicted by the PRISM **(D)** and CTRP **(E)** database, with point size inversely proportional to the p-value. The right panel compares the predicted drug responses between the high score and low score groups based on PRISM **(D)** and CTRP **(E)** database. **(F)** Comparison of predicted compound sensitivities across multi-omics subtypes. **(G)** Identification of most promising therapeutic compounds for patients with high risk scores according to the evidence from multiple sources. (ns, not significant; *, P ≤ 0.05; **, P ≤ 0.01; ***, P ≤ 0.001; ****, P ≤ 0.0001).

We analyzed the therapeutic opportunities for patients with different subtypes. The results showed that patients in the high C3 group exhibited the highest levels of cell cycle activity, DNA replication, hypoxia, and EGFR ligands compared to other subgroups (Figure 10A; Supplementary Table S7). This suggests that patients in the C3 group may be more sensitive to radiotherapy and EGFR-targeted therapy. In contrast, patients in the C2 group appeared to be less responsive to radiotherapy and EGFR-targeted therapy. However, we observed that the C2 group exhibited the highest levels of IDH1 pathway activity, while patients in the C1 group had the lowest levels of vascular endothelial growth factor A (VEGFA). This evidence indicates that different subtypes may involve distinct immunosuppressive mechanisms. Therefore, targeting these oncogenic pathways may provide promising therapeutic strategies for HCC patients.

For the estimation of drug response in patients with HCC, we constructed a drug response prediction model using the PRISM and CTRP dataset, which contains gene expression profiles and drug sensitivity data for hundreds of CCLs. The dataset includes a total of 1752 compounds (Figure 10B). We excluded compounds with NAs in more than 20% of the samples, as well as cell lines derived from hematopoietic and lymphoid tissues. Ultimately, 680 CCLs for 354 compounds in the CTRP dataset and 480 CCLs for 1,285 compounds in the PRISM dataset for subsequent analyses. The specific screening process is shown in Figure 10C. To identify drugs that are more effective in the high risk score group, we performed differential drug response analysis between the high risk score group (top 10%) and the low risk score group (bottom 10%) to identify compounds with lower estimated AUC values in patients with high risk scores. Subsequently, we conducted Spearman correlation analysis between AUC values and risk scores to select compounds with negative correlation coefficients (Spearman’s r < −0.35). Through these analyses, we identified four compounds (mitoxantrone, navitoclax, sirolimus, and volasertib) from the PRISM dataset and two compounds (fluvastatin and birinapant) from the CTRP dataset (Figures 10D, E). All of these compounds exhibited lower estimated AUC values in the high risk score group compared to the low risk score group and showed a negative correlation with the risk score (Figures 10D, E). These findings suggest that patients with high risk scores are more sensitive to these three candidate compounds than those with low risk scores. We further compared the potential drug sensitivity across different molecular subtypes. All candidate compounds exhibited the highest predicted sensitivity in patients from the C3 subgroup, followed by the C1 subgroup, while patients in the C2 subgroup consistently showed the lowest sensitivity to all compounds (Figure 10F).

To strengthen our findings, we conducted a series of analyses from multiple perspectives to further explore the therapeutic potential of the identified compounds in HCC. First, we systematically reviewed literature and databases including PubMed and DrugTarget to gather supporting experimental data and information on FDA approval status for the shortlisted compounds. The findings summarized in Figure 10G and Supplementary Table S8. We found that all the compounds had been reported to exhibit cytotoxic or inhibitory effects on HCC cells. Among them, sirolimus, mitoxantrone, and fluvastatin have been approved by the FDA for clinical use. Second, we evaluated the fold change in expression of drug target genes between tumor tissues and adjacent normal tissues, as well as between high risk and low risk patient groups. For each drug, the highest fold change among all its targets was selected as the representative value. A greater fold change was considered indicative of a higher therapeutic potential of the corresponding compound in HCC treatment. Notably, the targets of volasertib and mitoxantrone exhibited significantly elevated expression in tumor tissues, with especially high levels observed in high risk patient subgroups. Finally, we examined whether the shortlisted compounds have been clinically used for the treatment of HCC. We found that sirolimus and mitoxantrone have already been applied in the treatment of HCC patients.

## 4 Discussion

HCC is a highly heterogeneous disease, making it challenging to achieve a comprehensive understanding based solely on gene expression (Yang et al., 2019). Moreover, gene expression is finely regulated by multiple processes, including methylation, CNVs, and mutations (Oh et al., 2021). Integrative analysis of multi-omics data in HCC patients can provide deeper insights into tumor heterogeneity and its underlying regulatory mechanisms. However, most studies to date have primarily focused on single-omics approaches. Additionally, different clustering methods for multi-omics data are based on distinct principles, which further exacerbate the limitations of specific algorithms when applied in practice. To bridge this gap, our study incorporates ten state of the art clustering algorithms to identify three multi-omics subtypes with distinct prognostic, molecular, and TME characteristics. These newly defined subtypes may have important implications for the precision stratification and treatment of HCC patients.

In this study, we first focused on the multi-omics characteristics of the identified subtypes. We found that most methylation silencing events and large scale copy number variations occurred in C3 patients, while both C2 and C3 patients exhibited a high mutation burden. Notably, C2 patients were primarily characterized by CTNNB1 mutations, whereas C3 patients predominantly harbored TP53 mutations. This is consistent with previous reports that CTNNB1 and TP53 mutations exhibit a mutually exclusive pattern in HCC (Liang et al., 2021). Previous studies have shown that CTNNB1 mutations are characteristic alterations associated with the immune excluded phenotype (Pinyol et al., 2019), linked to WNT/β-catenin pathway activation (Xu et al., 2022) and a low response rate to immunotherapy (Harding et al., 2018). Our results also confirmed this observation. Compared to C1 and C3 patients, C2 patients exhibited significantly lower levels of MHC-I, MHC-II, immunosuppressive factors and immunostimulatory factors, indicating a less active immune response in this group. Studies have shown that TP53 mutations in HCC patients are significantly associated with elevated serum alpha fetoprotein (AFP) levels (>300 ng/mL), larger tumor size (>5 cm), higher tumor grade (III/IV), and an increased risk of mortality (Woo et al., 2010a). Our results also corroborated this finding, as the C3 group, predominantly characterized by TP53 mutations, was significantly associated with advanced stage, higher T stage, elevated AFP levels, female gender, and poorer prognosis. Compared to C2 and C3 patients, C1 patients exhibited fewer significant CNV and methylation silencing events, with only a few notable mutations occurring at very low frequencies. However, their MHC-I, MHC-II, and immunostimulatory factors indicated a more active immune system, while their levels of immunosuppressive factors were relatively lower than those in C3. Additionally, C1 patients had the lowest CNV burden, methylation burden, and mutation burden, which may suggest that C1 tumors have lower heterogeneity and invasiveness. These factors may contribute to the relatively favorable prognosis observed in C1 patients. These immune related characteristics may contribute to the relatively favorable prognosis observed in C1 patients. Overall, our multi-omics classification system effectively distinguishes the multi-omics characteristics of HCC patients and shows significant associations with clinical features. This classification may facilitate clinical application and patient stratification, highlighting its potential clinical utility.

Beyond clinical and molecular characteristics, we also focused on TME features. Our results showed that, compared to C1 and C2 patients, C3 patients exhibited a significantly lower proportion of hepatocytes and a higher proportion of cholangiocytes and macrophages. This is a particularly intriguing finding, suggesting that molecular characteristics identified through multi-omics analysis not only influence clinical features and prognosis but also shape the spatial composition and structure of the TME.

First, we further performed a subgroup analysis of macrophages and found that Macro_2_SPP1 exhibited the highest M2 polarization score and angiogenesis score, along with a lower phagocytosis score, and was significantly associated with poor prognosis. Previous studies have shown that SPP1 expressing Tumor Associated Macrophages (TAMs) are primarily enriched in AFP positive HCC and are characterized by reduced phagocytic function but enhanced pro-angiogenic capacity, which aligns with our findings (He et al., 2023). Although Macro_2_SPP1 did not show a statistically significant difference in distribution among C1, C2, and C3 patients, there was a noticeable trend toward higher levels in the C3 group, which also represents the AFP-high subtype. Additionally, studies have demonstrated that SPP1 macrophages can bind to CD44 on the surface of T cells, thereby suppressing their anti-tumor function. Targeting and blocking the SPP1/CD44 pathway has been shown to restore T cell function and inhibit tumor growth, suggesting that this pathway may serve as a promising therapeutic target for HCC (He et al., 2023). Additionally, our study identified a macrophage subset characterized by high C1QA expression, which exhibited the highest phagocytosis score and was significantly less abundant in C3 patients, yet strongly associated with better prognosis. Recent study has shown that this macrophage subset is linked to gene signatures associated with a favorable response to immune checkpoint therapy and primarily functions in phagocytosis and antigen presentation (Liu et al., 2022). Moreover, Macro_1_C1QA was predominantly located at the tumor periphery, whereas Macro_2_SPP1 was enriched in the tumor core, suggesting that these two macrophage subsets may be involved in shaping distinct tumor ecological niches.

Regarding cholangiocytes, we observed a striking phenomenon in which their proportion was significantly elevated in the C3 group. Study has shown that approximately 28% of HCC cases express cholangiocytic markers CK7 and/or CK19 (Durnez et al., 2006). Woo et al. found that approximately 20% of HCCs share gene expression characteristics with cholangiocarcinoma and exhibit stem cell-like features (Woo et al., 2010b). Kim et al. reported that CK19 expression in HCC was significantly associated with microvascular invasion, fibrous stroma, and poor clinical outcomes (Kim et al., 2011). Collectively, these studies suggest that a proportion of HCCs display cholangiocytic characteristics, which are linked to worse clinical outcomes. Therefore, we conducted a detailed analysis of cholangiocyte subpopulations and found that Cholangio_0_TCF4, Cholangio_2_KRT19, and Cholangio_4_S100P were significantly enriched in C3 patients, whereas Cholangio_1_AGXT was more likely to be enriched in C1 and C2 patients. Thus, the increased proportion of cholangiocytes observed in C3 is likely attributable to the enrichment of Cholangio_0_TCF4, Cholangio_2_KRT19, and Cholangio_4_S100P. Our findings suggest that high levels of Cholangio_1_AGXT are associated with better prognosis, while Cholangio_4_S100P is linked to worse prognosis. Studies have demonstrated that cholangiocytes possess a high proliferative capacity and commonly proliferate in response to liver injury and inflammation (Alvaro et al., 2007). During this process, proliferating cholangiocytes secrete a range of cytokines, neuropeptides, and growth factors, contributing to cross-talk with other cell types within the microenvironment. We hypothesize that the S100P^+^ cholangiocytes observed in HCC may represent an aberrantly proliferative and activated population induced by tumor microenvironmental stimuli. Overall, by analyzing single-cell sequencing data, we characterized the heterogeneity of cholangiocytes in HCC and evaluated their prognostic significance. Importantly, we successfully identified a subpopulation of S100P^+^ cholangiocytes associated with poor prognosis in HCC, which may represent a novel and biologically relevant cell subset worthy of further investigation.

Through single-cell interaction analysis, we identified that APOA1-TREM2 and APOA2-TREM2 play critical roles in the crosstalk between hepatocytes and macrophages. TREM2 has attracted increasing attention in recent years, as emerging evidence suggests its close association with immunosuppressive macrophage phenotypes (Molgora et al., 2023). TREM2^+^ TAMs have been shown to suppress T cell activity (Katzenelenbogen et al., 2020). Moreover, the enrichment of TREM2^+^ macrophages has been correlated with poor response to anti-PD-1 therapy, indicating that this cell subset may contribute to immunotherapy resistance (Molgora et al., 2020). Targeting TREM2 may therefore represent a promising strategy to enhance the efficacy of anti-PD-1 treatment. Studies on ApoA1 and ApoA2 in HCC remain limited. Existing evidence indicates that APOA1 expression is downregulated during HCC progression and is significantly positively associated with the prognosis of HCC patients (Xu et al., 2025). In contrast, high APOA2 expression in HCC may sustain or promote PD-L1 expression, thereby enhancing tumor immune evasion (Qi et al., 2024). However, studies investigating the interaction between APOA1/2 and TREM2 are currently lacking. Nevertheless, this represents a highly promising avenue for future research.

We also observed that the VTN-PLAUR axis plays an important role in the interaction between cholangiocytes and macrophages. Studies have shown that PLAUR can promote tumor metastasis by mediating plasminogen activation and extracellular matrix degradation (Laurenzana et al., 2017). High expression of PLAUR has been reported in various cancers and is generally associated with poor survival and prognosis (Gilder et al., 2018; Hildenbrand and Schaaf, 2009; Boonstra et al., 2011). Aberrant expression of PLAUR in tumors may also contribute to increased infiltration of TAM (Lindsten et al., 2017). However, the role of PLAUR in HCC and macrophages remains relatively underexplored. Research on VTN remains limited. Existing studies have identified VTN as a candidate biomarker for the development of HCC in patients with HCV related cirrhosis (Ferrín et al., 2014). However, the relationship between VTN and macrophages in the context of HCC has not been well investigated. Overall, we identified ligand–receptor pairs that play key roles in the interactions among hepatocytes, cholangiocytes, and macrophages. Although there is currently insufficient research to fully elucidate their roles in HCC, existing evidence suggests that these molecules may have important potential significance.

Using machine learning methods, we successfully identified six potentially effective drugs (mitoxantrone, navitoclax, sirolimus, volasertib, fluvastatin and birinapant) for high risk HCC patients. Among these, we found that the molecular targets of mitoxantrone and volasertib were significantly overexpressed in high risk patients. In addition, both mitoxantrone and sirolimus have already been used in the clinical management of HCC. Therefore, we focused our subsequent analyses on mitoxantrone, sirolimus, and volasertib.

Mitoxantrone, a synthetic anthracenedione derivative, has been shown to exhibit antitumor activity against various cancer cell types both *in vitro* and *in vivo*. Its antitumor effects may involve multiple mechanisms, such as stabilizing topoisomerase II-DNA cleavage complexes, thereby preventing the religation of DNA strand breaks, and generating free radicals, among others (Wiseman and Spencer, 1997). Mitoxantrone has demonstrated efficacy comparable to standard induction and salvage therapy regimens in the treatment of advanced breast cancer, non-Hodgkin’s lymphoma, and acute non-lymphocytic leukemia (Wiseman and Spencer, 1997). A few clinical studies and retrospective reports have explored the use of mitoxantrone as part of combination chemotherapy regimens (often in conjunction with cisplatin, 5-fluorouracil (5-FU), or doxorubicin) for the treatment of advanced HCC (Liang et al., 2011; Yang et al., 2004; Ikeda et al., 2005). However, given that many HCC patients suffer from impaired liver function, the use of mitoxantrone poses a relatively high risk of hepatotoxicity, which has limited its widespread clinical application. To overcome these challenges, recent studies have attempted to encapsulate mitoxantrone in nanoparticle formulations, liposomes, or targeted delivery systems to enhance tumor specific accumulation in HCC and reduce systemic toxicity (Lam et al., 2018).

Sirolimus, also known as Rapamycin, is a macrolide immunosuppressant that primarily inhibits the mTOR signaling pathway to block the activation of T cells and B cells. It was initially approved for the prevention of organ rejection in kidney transplant patients (Nguyen et al., 2019). Multiple studies have since demonstrated that the use of sirolimus after liver transplantation in patients with HCC is associated with reduced recurrence rates and recurrence related mortality, as well as prolonged recurrence free survival and overall survival (Grigg et al., 2019; Menon et al., 2013).

Volasertib is a Polo-like kinase one inhibitor primarily used to disrupt cell division. It has been evaluated in clinical trials for cancers such as acute myeloid leukemia (Hao and Kota, 2015). Our results showed that the mRNA expression level of PLK1, the molecular target of Volasertib, was elevated in tumor tissues, particularly in high risk HCC patients. This suggests that Volasertib may represent a promising therapeutic compound for this patient subgroup. Moreover, a recent study combining deep learning approaches with cellular experiments has provided further evidence supporting the antitumor efficacy of Volasertib in HCC (Zhang et al., 2025). Overall, these novel potential compounds may provide new research directions for the treatment of HCC.

## 5 Conclusion

In this study, we identified three distinct molecular subtypes in HCC patients using multi-omics consensus clustering analysis. These subtypes exhibited significant prognostic differences and distinct clinical and molecular characteristics, potentially refining the molecular classification of HCC. Additionally, we thoroughly investigated the TME differences among these subtypes, revealing a lower proportion of hepatocytes and a higher proportion of macrophages and cholangiocytes in patients classified as subtype C3. Furthermore, we employed machine learning methods to construct a prognostic model for HCC patients and identified novel potential compounds for high risk patients. By integrating multi-omics datasets with advanced computational approaches, this study provides a theoretical foundation for improving early diagnosis and enabling precision treatment strategies for HCC patients.

## Data Availability

The datasets presented in this study can be found in online repositories. The names of the repository/repositories and accession number(s) can be found in the article/supplementary material.
