# Supplementary figures and images for: Multi-omics and single-cell approaches reveal molecular subtypes and key cell interactions in hepatocellular carcinoma
Source: Front Pharmacol. 2025 May 22;16:1605162. doi: 10.3389/fphar.2025.1605162 (PMC12138379; doi:10.3389/fphar.2025.1605162)

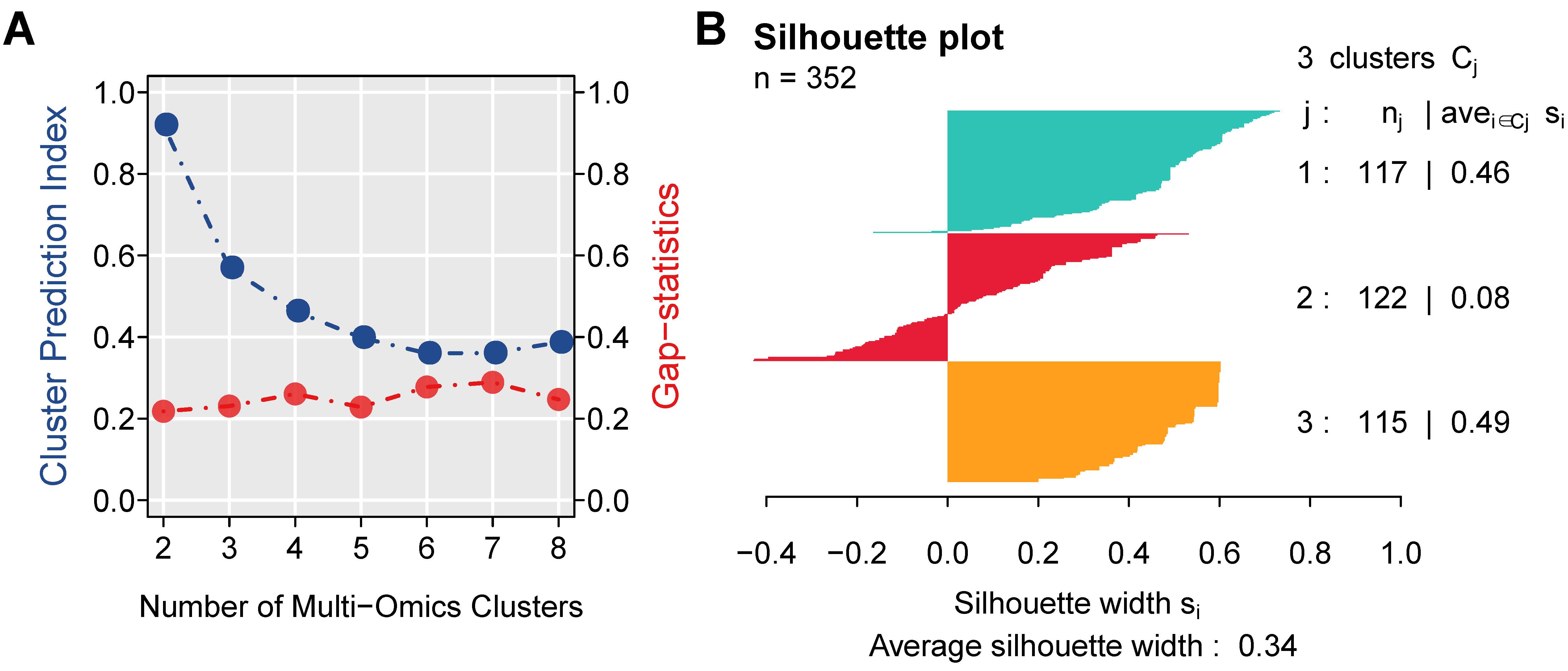

Supplement: Supplementary file 2 [file Image3.jpeg]

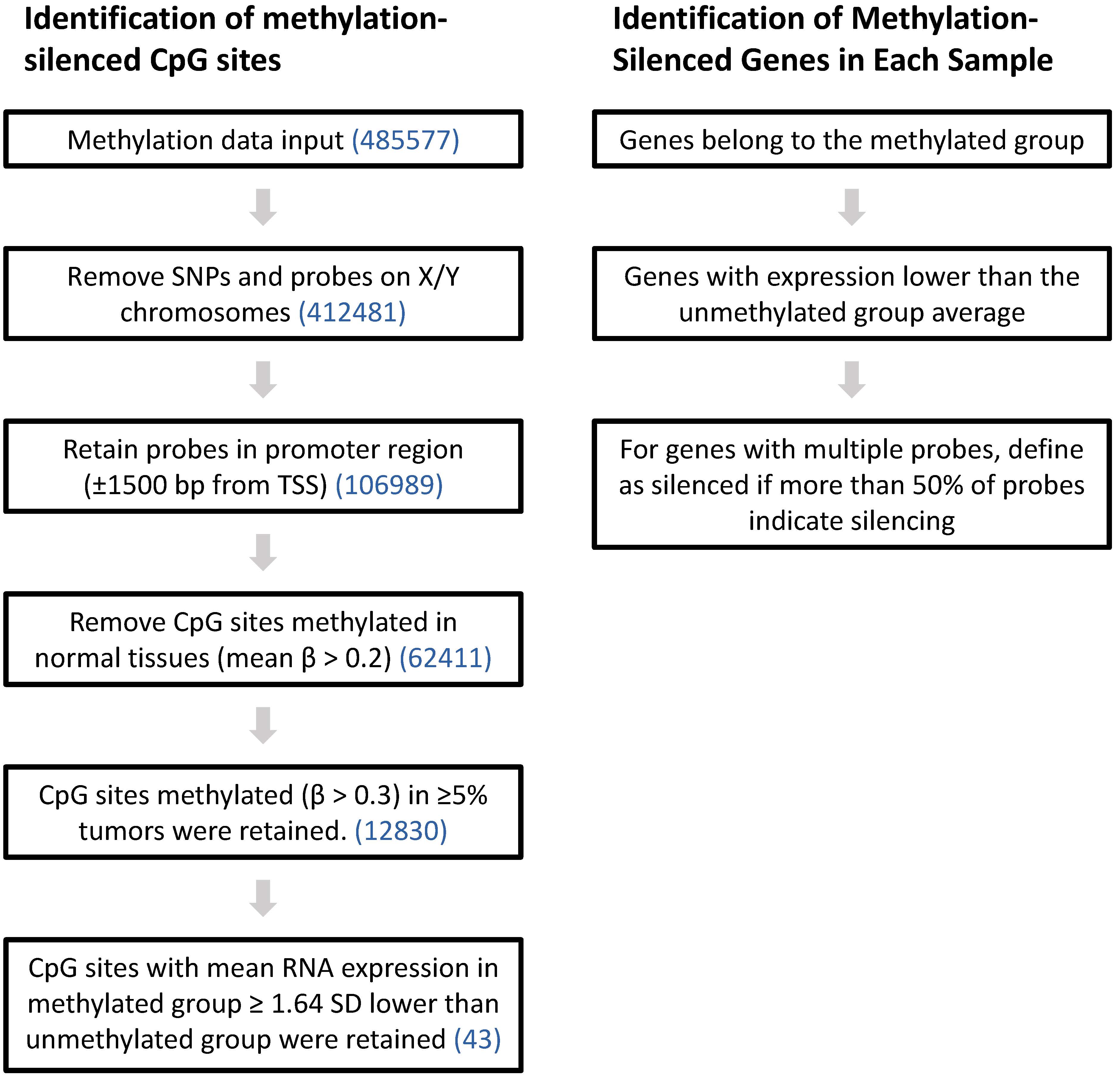

Supplement: Supplementary file 4 [file Image1.jpeg]

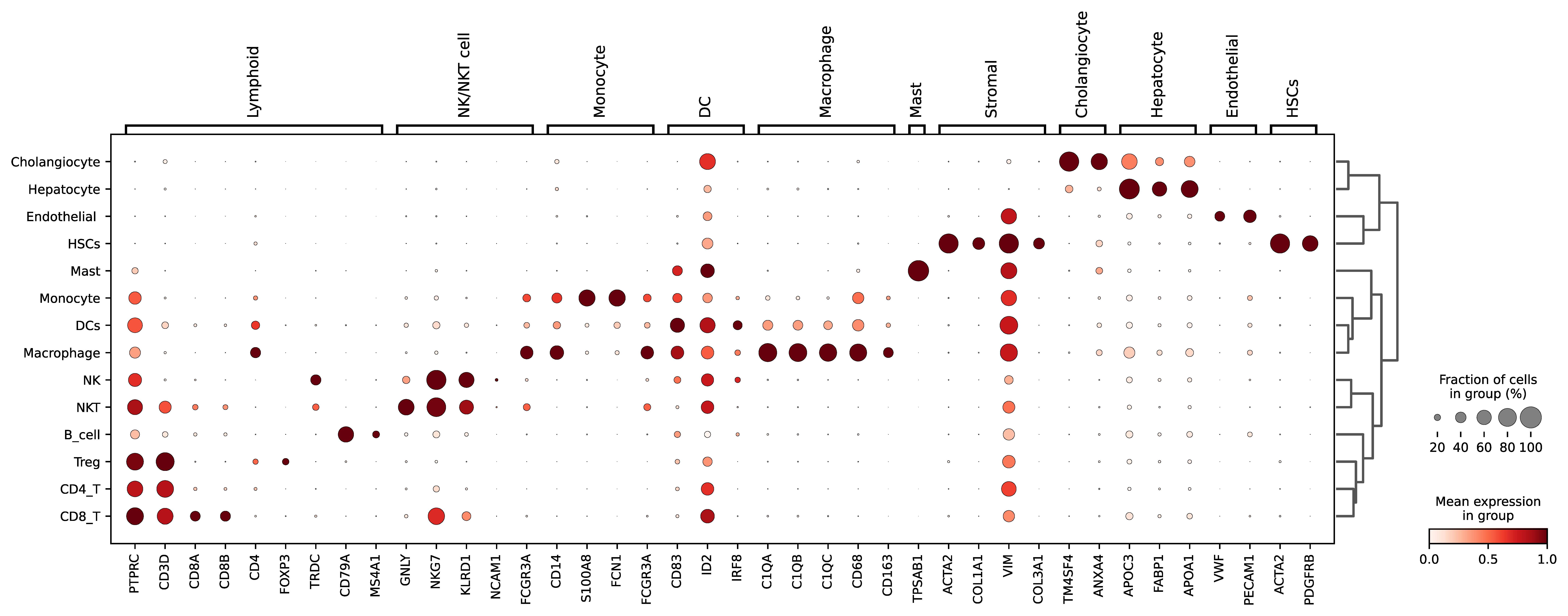

Supplement: Supplementary file 5 [file Image4.jpeg]

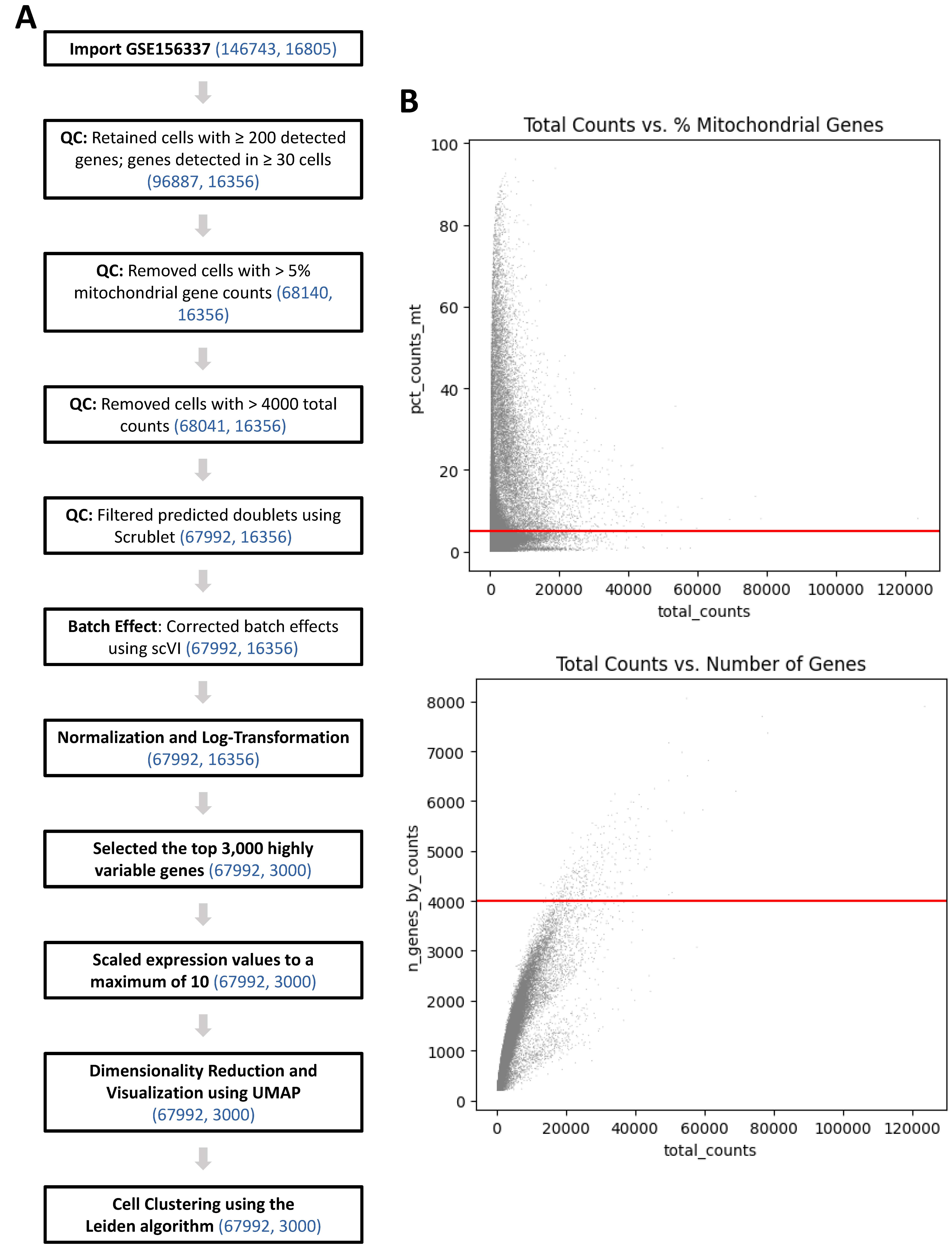

Supplement: Supplementary file 6 [file Image2.jpeg]

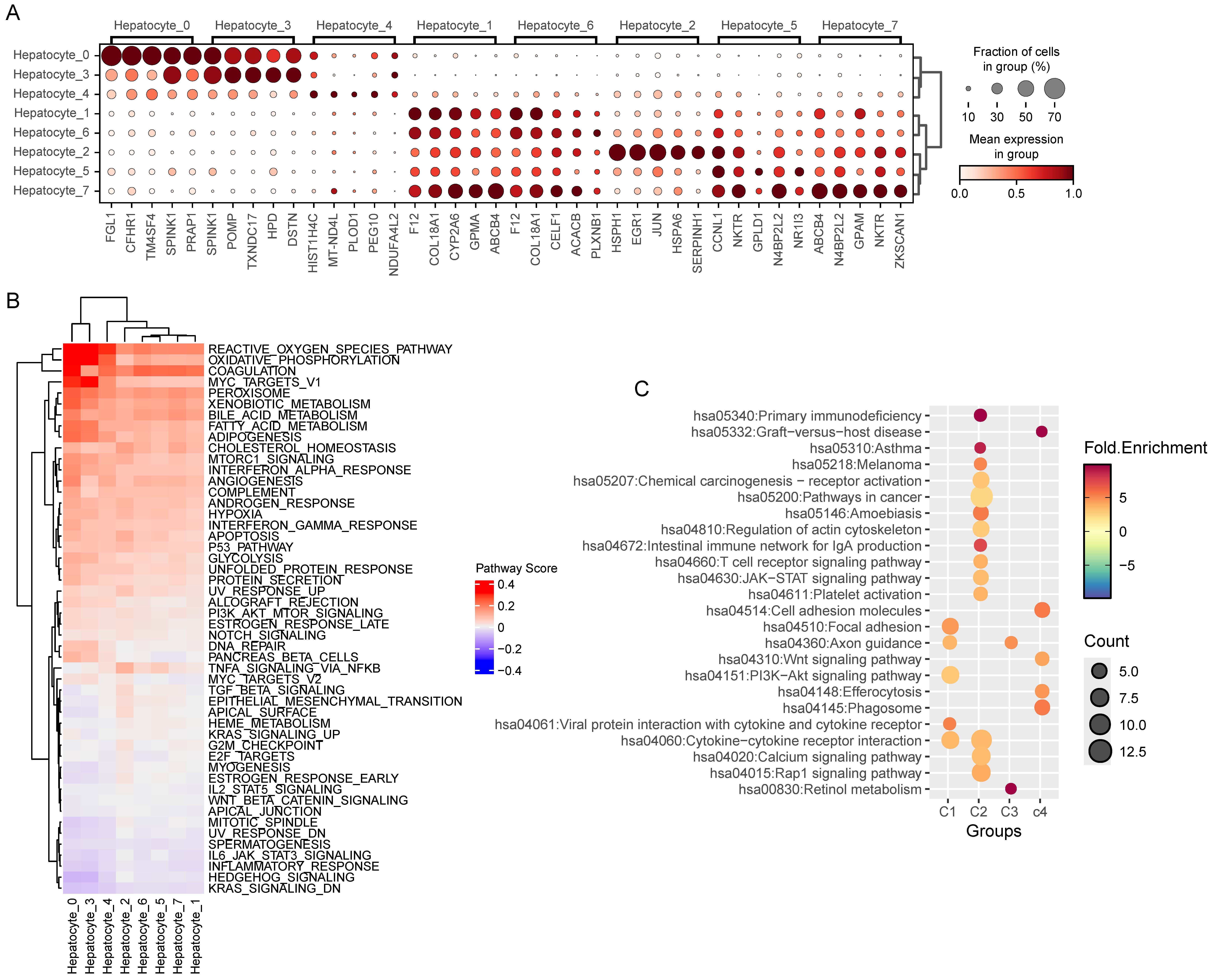

Supplement: Supplementary file 7 [file Image5.jpeg]

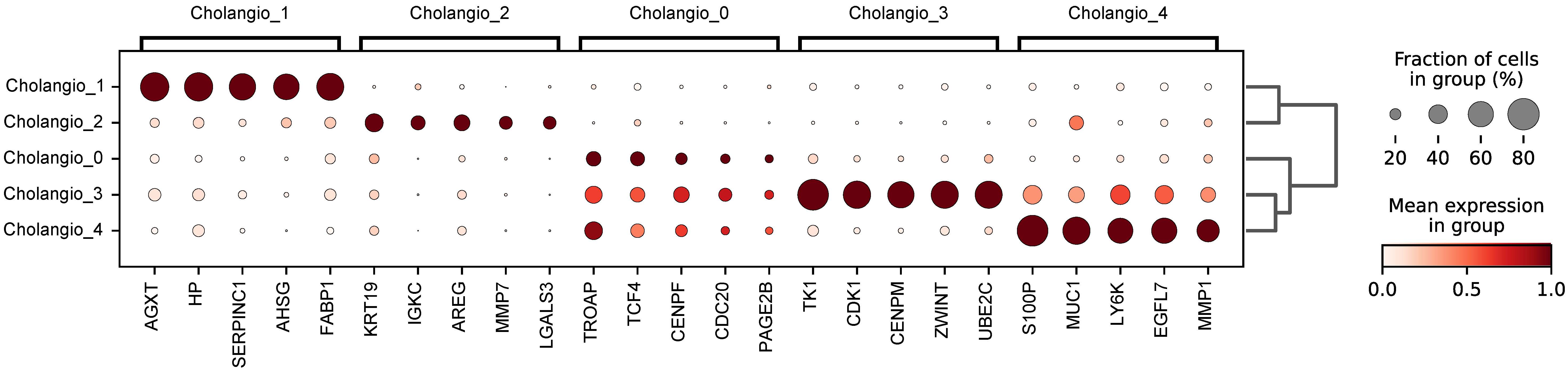

Supplement: Supplementary file 14 [file Image6.jpeg]
